# Membrane-mediated protein interactions drive membrane protein organization

**DOI:** 10.1038/s41467-022-35202-8

**Published:** 2022-11-30

**Authors:** Yining Jiang, Batiste Thienpont, Vinay Sapuru, Richard K. Hite, Jeremy S. Dittman, James N. Sturgis, Simon Scheuring

**Affiliations:** 1 Biochemistry & Structural Biology, Cell & Developmental Biology, and Molecular Biology (BCMB) Program, Weill Cornell Graduate School of Biomedical Sciences, 1300 York Avenue, New York, NY 10065 USA; 2 Weill Cornell Medicine, Department of Anesthesiology, 1300 York Avenue, New York, NY 10065 USA; 3 Laboratoire d’Ingénierie des Systèmes Macromoléculaires (LISM), Unité Mixte de Recherche (UMR) 7255, Centre National de la Recherche Scientifique (CNRS), Aix Marseille Université, Marseille, France; 4 Structural Biology Program, Memorial Sloan Kettering Cancer Center, 1275 York Avenue, New York, NY 10065 USA; 5 Physiology, Biophysics, and Systems Biology (PBSB) Program, Weill Cornell Graduate School of Biomedical Sciences, 1300 York Avenue, New York, NY 10065 USA; 6 Weill Cornell Medicine, Department of Biochemistry, 1300 York Avenue, New York, NY 10065 USA; 7 Weill Cornell Medicine, Department of Physiology and Biophysics, 1300 York Avenue, New York, NY 10065 USA; 8 Kavli Institute at Cornell for Nanoscale Science, Cornell University, Ithaca, NY 14853 USA

**Keywords:** Membrane structure and assembly, Single-molecule biophysics

## Abstract

The plasma membrane’s main constituents, i.e., phospholipids and membrane proteins, are known to be organized in lipid-protein functional domains and supercomplexes. No active membrane-intrinsic process is known to establish membrane organization. Thus, the interplay of thermal fluctuations and the biophysical determinants of membrane-mediated protein interactions must be considered to understand membrane protein organization. Here, we used high-speed atomic force microscopy and kinetic and membrane elastic theory to investigate the behavior of a model membrane protein in oligomerization and assembly in controlled lipid environments. We find that membrane hydrophobic mismatch modulates oligomerization and assembly energetics, and 2D organization. Our experimental and theoretical frameworks reveal how membrane organization can emerge from Brownian diffusion and a minimal set of physical properties of the membrane constituents.

## Abstract (web summary)

High-Speed Atomic Force Microscopy movies of membrane proteins — diffusing and interacting in bilayers of controlled thickness — allow the determination of membrane-mediated membrane protein interaction energetics.

## Introduction

In an amended version of the fluid mosaic model^1,2^, the membrane is not a passive medium but plays an active role modulating membrane protein function and organization through its physical properties^3,4^. Changes in membrane protein function that depend on membrane properties have been measured experimentally using approaches such as electrophysiology and fluorescence-based vesicle transport assays^5,6^. In contrast, the direct experimental study of membrane-mediated membrane protein oligomerization and assembly remains challenging.

The two-dimensional (2D) organization of a biological membrane would be random if the interaction energies between all components were of the order of *k*_B_*T*^2^. In reality, cell membranes and their constituent membrane proteins display a non-random organization. Fluorescence microscopy and biochemical observations have reported lipid-protein rafts^7,8^, functional domains^9,10^, and membrane protein supercomplexes^11,12^, clear signatures of non-randomness of biological membranes. In eukaryotic cells, both membrane components, e.g., phospholipids and cholesterol, and the peripheral environment, e.g., cytoskeleton and extracellular matrix, contribute to non-random membrane organization^13^. Peripheral interactions tether membrane molecules and serve as lateral diffusing barriers^14^, while the membrane is the medium for molecules to interact and its influence can be studied in a controlled way. To the best of our knowledge, no active process intrinsic to the membrane is known to steer and place membrane proteins in membranes. Thus, the question is: What drives membrane organization? First, membrane protein interactions can be protein-mediated, meaning that the two partner molecules make direct protein–protein contact, or interact via a third protein that holds them together. In this case, strong interactions, e.g., hydrogen bonds, ionic- and dipole-dipole interactions, can be formed between the partner molecules. Second, membrane protein interactions can be membrane-mediated, where mainly hydrophobic amino acid residues on a membrane protein surface are exposed to the hydrophobic phospholipid bilayer core. As a result, none of the above-mentioned strong interactions can be formed. In this case, sufficient energy, i.e., several *k*_B_T, must be generated from weak hydrophobic interactions between lipids and membrane proteins as well as intrinsic membrane physical properties. In the past decades, numerous theoretical and computational studies have predicted a key role of the membrane mechanics, e.g. thickness, stiffness, curvature, and tension, in these interactions^3,15–22^. While strong hydrophilic interactions via cytoplasmic and extracellular domains and weak hydrophobic interactions can coexist, an interesting aspect of membrane-mediated interactions is their long range. Indeed, membrane proteins sense each other through the membrane over distances up to ~10 nm^15^. As a result, attractive and repulsive membrane-mediated long-range interactions drive protein positioning and organization before local electrostatic interactions can form between proteins at short distances on the order of ~2 nm. Therefore, investigating membrane-mediated interactions is crucial for understanding membrane organization more generally.

While circular dichroism^23^, single-molecule fluorescence microscopy^24^, fluorescence correlation spectroscopy (FCS)^25^, and Förster resonance energy transfer (FRET)^26^ have been employed to study membrane protein interactions and have provided invaluable observations that informed theory, these approaches are more indirect, make use of labels and/or are resolution limited. Here, we report an experimental design employing high-speed atomic force microscopy (HS-AFM)^27,28^ to directly visualize and quantify membrane-mediated interactions of unlabeled membrane proteins at high spatial and temporal resolution: We use the *Escherichia coli* water channel Aquaporin-Z (AqpZ) and synthetic lipids of defined hydrocarbon tail length as an experimental model system to study the oligomerization and interaction energies of membrane proteins as a function of the bilayer thickness in which they are embedded. The experimental system is well-defined: (i) AqpZ is solved to high-resolution by X-ray crystallography^29^, providing details about the AqpZ structure and its hydrophobic thickness. (ii) An AqpZ-W14A mutant exposes surfaces to the membrane akin the AqpZ-WT tetramer, but has destabilized protomer interfaces^30^, enabling us to study both the protein assembly and oligomerization processes. (iii) The thickness of the synthetic purified lipids used here have been resolved by small-angle X-ray diffraction^31^, providing precise control and knowledge of the membrane environment in which the membrane-mediated protein interactions are measured. Finally, (iv) the HS-AFM movies provide unique direct structural and dynamic data exploitable for quantitative analysis.

## Results

### Experimental design to study membrane-mediated protein interactions

**Fig. 1:**
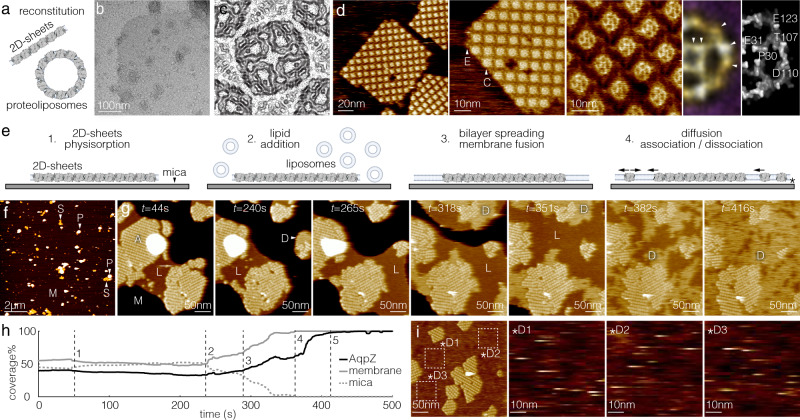
Sample characterization and experimental strategy to study membrane-mediated protein interactions at the single-molecule level. **a** Sample: Reconstitution at lipid-to-protein ratio (LPR) of 0.1 (w:w) results in 2D-crystalline AqpZ proteo-liposomes and sheets. **b**–**d** Sample characterization. **b** Negative stain electron microscopy (EM): Tetragonal packing of AqpZ in a 2D-sheet; Supplementary Fig. 1. **c** Cryo-EM 2D-crystallography: Projection map at 4 Å resolution (1 unit cell, side length: 95 Å; Supplementary Fig. 2). **d** HS-AFM images at three different magnifications (Left to right: 0.5 nm/pixel, 0.33 nm/pixel, and 0.17 nm/pixel). E: Extracellular surface. C: Cytoplasmic surface. Right: LAFM map and surface representation of the X-ray structure PDB 3NKC. Surface protruding amino acids are labeled in the structure (arrowheads in LAFM map). **e** Experimental strategy to study membrane-mediated protein interactions: 1. Sample physisorption to the mica HS-AFM support. 2. Addition of liposomes of lipids with hydro-carbon chain length C14, C16, C18, C20 (Supplementary Fig. 4). 3. Lipid spreading on the mica leads to fusion of the free bilayer with AqpZ sheets. 4. Equilibrium membrane protein interaction dynamics: Diffusion, association, and dissociation. Asterisk: buffer layer between mica surface and lipid bilayer due to electrostatic shielding of surface charges on mica and protein allows protein diffusion on the atomically flat mica surface. **f** AFM overview. The sample covers <5% of the surface. M: Mica. P: Proteo-liposomes. S: 2D-sheets. **g** HS-AFM movie frames (Supplementary Movie 1) of the membrane fusion experiment (DOPC). M: Mica. A: AqpZ array. L: Lipid bilayer. D: Diffusing AqpZ. **h** Analysis of the membrane fusion process in (**g**). (1) Lipid addition. (2) Bilayer spreading and membrane fusion. 3: Onset of AqpZ diffusion. 4: 100% membrane coverage. 5: 100% coverage of the membrane by diffusing molecules. **i** Diffusion in the membrane regions indicated by dashed squares labeled D1, D2 and D3 (left) (Supplementary Movie 2). Right: Enlarged and contrast enhanced images at slightly increased imaging force of the diffusion fields D1, D2, D3. Similar results as in (**b**), (**d**), (**f**), (**g**), and (**i**) were observed in all samples/experiments from all biological replica. Schematics in (**a**) and (**e**) were generated using Biorender.com.

To study membrane-mediated protein interactions, we reconstituted AqpZ-W14A into a phospholipid bilayer consisting of 1,2-dioleoyl-sn-glycero-3-phosphocholine (DOPC), 1,2-dioleoyl-sn-glycero-3-phosphoethanolamine (DOPE) and 1,2-dioleoyl-sn-glycero-3-phospho-L-serine (DOPS) at ratio 8:1:1 (w:w:w) (Fig. 1a). The membrane-embedded AqpZ molecules formed 2D crystalline arrays, either in sheets or in proteo-liposomes, due to reconstitution at very low lipid-to-protein ratio (LPR) of 0.1 (w:w; ~20 lipid molecules per AqpZ tetramer) (Fig. 1b, Supplementary Fig. 1). To get detailed insights into the sample morphology, we solved a projection map of the AqpZ 2D-arrays to 4 Å resolution using cryo-electron microscopy (Cryo-EM; Fig. 1c, Supplementary Fig. 2). The Cryo-EM analysis of the 2D-array revealed *p*42_1_2 plane symmetry group, where each AqpZ contacted four AqpZ in the opposite orientation^32,33^, and the protein coverage in the 2D-arrays was ~80%. We imaged the AqpZ 2D-arrays using tapping-mode HS-AFM at various magnifications (Fig. 1d). In HS-AFM, the majority of the observed AqpZ arrays had a size of ~200 nm in diameter and allowed the extracellular and the cytoplasmic sides of AqpZ to be resolved (Fig. 1d, arrowheads E, C). From high-resolution HS-AFM images, we calculated a localization AFM map (LAFM)^34^ of the extracellular AqpZ surface, in which details of surface protruding residues were resolved (Fig. 1d, right).

**Fig. 2:**
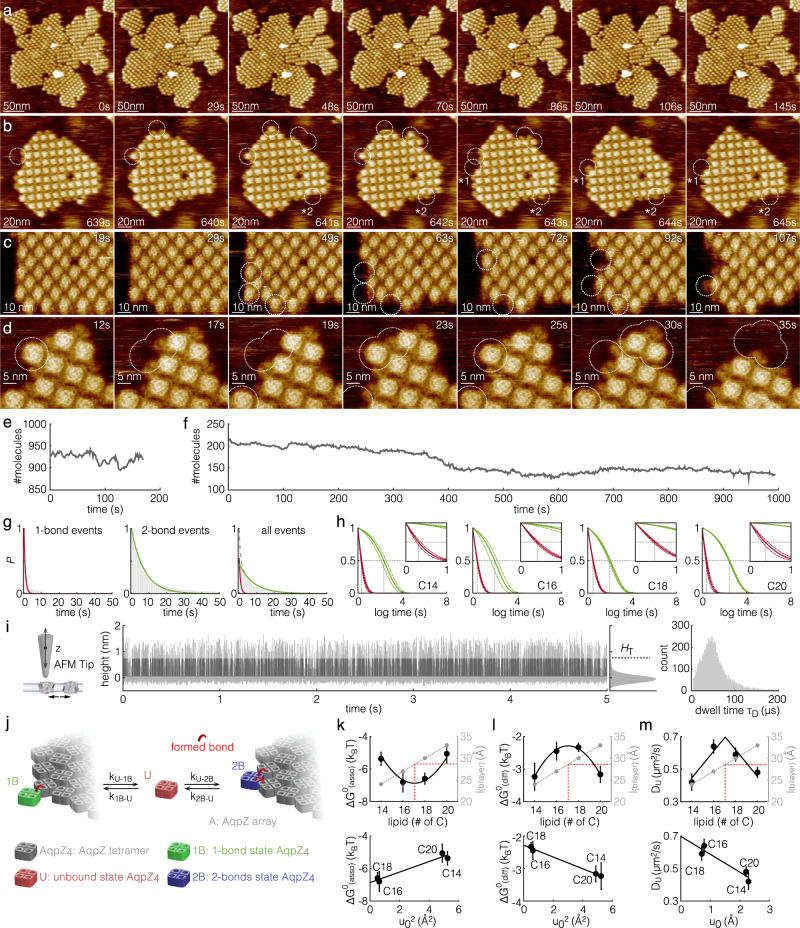
The hydrophobic mismatch between protein and phospholipid bilayer impacts membrane protein interactions and diffusion. **a**–**d** HS-AFM movie frames (Supplementary Movies 3–6) of single-molecule association-dissociation dynamics at the edges of AqpZ arrays in a C18 membrane (image parameters: (**a**) 1.0 nm/pixel, (**b**) 0.5 nm/pixel, (**c**) 0.33 nm/pixel, and (**d**) 0.17 nm/pixel). Dashed outlines: association-dissociation events. Asterisk 1: one-bond event. Asterisk 2: two-bond event. These image series have been acquired 40 min (**a**), 2 h (**b**), 40 min (**c**), and 15 min (**d**) after lipid vesicle addition and continuous bilayer formation. **e**, **f** Number of molecules vs time of Supplementary movies 3 and 4, respectively (panels (**a**) and (**b**)). **g** Dwell-time distributions of association-dissociation events in a C18-membrane. One-bond (left, (*n* = 246) and two-bond (center, *n* = 549) events were fitted separately based on imaging knowledge using one exponential, or collectively using two exponentials (right, *n* = 1096). **h** Normalized fittings of all events in C14 (*n* = 308, 3 replicas), C16 (*n* = 761, 3 replicas), C18 (*n* = 1096, 3 replicas) and C20 (*n* = 288, 3 replicas) membranes (as indicated). Insets: Detail views of the fast exponential decay. Thick lines: averages. Thin lines: ±s.e. **i** HS-AFM height spectroscopy (HS-AFM-HS). Left: Schematic of HS-AFM-HS principle: The tip is at a fixed location monitoring molecular diffusion events. Middle: HS-AFM-HS height-time trace. Light gray: raw data. Dark gray: diffusion events, threshold height *H*_T_ = 5std above mean of the height distribution next to the trace. The 0 nm height level was set to the membrane surface. Right: Distribution of event dwell times τ_D_. **j** Model of the membrane-mediated protein interactions where a diffusing molecule U can engage a 1B (one-bond) or 2B (two-bond) interaction with the array. **k** Association energy (*ΔG*^*0*^_asso_), defined as the energy difference between states U and B, (**l**) energy difference between states 1B and 2B (*ΔG*^*0*^_*diff*_), and (**m**) diffusion coefficient (*D*_*U*_), as functions of the acyl-chain length (top) and hydrophobic mismatch (*u*_*0*_), or its squared value (*u*_0_^2^) (bottom). *l*_bilayer_: Hydrophobic thickness of the membrane. The hydrophobic mismatch is calculated as *u*_0_ = *0.5*|*l*_bilayer_ *– l*_AqpZ_|, where *l*_AqpZ_ is the hydrophobic thickness of AqpZ (~28.6 Å, Supplementary Fig. 5, dashed red lines in the top panels). Solid curves are quadratic and linear fits to the data points. Statistics (mean±s.e.) in (**k**) and (**m**) are determined from three biological replica, in each condition. Statistics (mean±s.e.) in (**i**) is relevant to the statistics in (**h**), according to Eq. (4).

The AqpZ arrays serve as model membrane protein assemblies to study membrane-mediated protein interactions upon controlled lipid addition. In a typical experiment, membrane-embedded AqpZ is sparsely distributed on the mica HS-AFM sample support (Fig. 1e, step 1, Fig. 1f). The arrays initially covered <5% of the entire mica (Fig. 1f), meaning that the lipid coverage provided by the sample represents <1% of the surface. This is important for the ensuing of the experiment, in which vesicles of defined lipid composition are supplemented to the HS-AFM fluid cell, pure DOPC liposomes in the presented experiment (Fig. 1e, step 2, Fig. 1g, *t* = 44 s). The added lipids spontaneously dispersed across the mica surface and fused with existing membrane patches to cover the entire imaging area with a lipid bilayer (Fig. 1e, step 3, Fig. 1g, *t* = 240 s to *t* = 351 s). AqpZ dissociated from the edges of the protein arrays and diffused into the newly formed lipid bilayer, rapidly reaching a new dynamic equilibrium state comprising a mixture of AqpZ protein arrays and freely diffusing molecules (Fig. 1e, step 4, Fig. 1g, *t* = 382 s to *t* = 416 s). The presence of rapidly diffusing AqpZ was revealed by the increased average height of the bilayer areas as compared to empty bilayer. These areas (Fig. 1g, labeled D) i.e. the diffusing molecules, had a height of ~1 nm above the membrane level, slightly lower than the extracellular face of stable molecules. This is expected as the average height of transient molecules comprises frequent detections of the edges of fast diffusing molecules, thus recording a lower height than that of a stable molecule. In the example experiment (Fig. 1g, Supplementary Movie 1), vesicle addition (Fig. 1h, step 1) initiated bilayer spreading after ~180 s (Fig. 1h, step 2) which covered the surface within ~120 s (Fig. 1h, steps 2–4), while membrane protein diffusion started ~50 s after the first occurrence of membrane fusion (Fig. 1h, step 3) and equilibrated within ~50 s after complete membrane formation (Fig. 1h, steps 3–5). At this stage, the entire surface was covered with a single protein-lipid layer and no vesicular structures were found (see Fig. 1g, *t* = 265 s to *t* = 318 s). Zooming into membrane regions in HS-AFM imaging mode at slightly increased imaging force revealed diffusing molecules as transient streaks in scan lines (Fig. 1i, Supplementary Movie 2). Quantitative characterization of the diffusing molecules is possible using HS-AFM height spectroscopy^35^, as shown in Fig. 2.

The experimental design described here (Fig. 1) combined with the high spatio-temporal resolution of HS-AFM, ~0.5 nm/pixel at 1 frame/s in the exemplified experiment, allows us to study membrane-mediated protein interactions at the single-molecule level (Supplementary Movie 1). Importantly, given that i) the initial sample surface coverage was <5% (Fig. 1f), ii) the reconstitution was performed at LPR 0.1, and iii) the protein covers ~80% in the 2D-arrays as reported by the cryo-EM map (Fig. 1c, Supplementary Fig. 2), we know that the subsequently added vesicles contribute >99% of the lipid surface coverage in the experiments.

### Experimental quantification of membrane-mediated protein interactions

Under the experimental conditions described here, the AqpZ-membrane system reached equilibrium after about 6 min: The bilayer covered the entire sample surface, the bilayer fused with the pre-existing protein patches, and molecules freely diffused throughout the bilayer (Fig. 1h, step 5). We waited for another >15 min and recorded single-molecule membrane-mediated association-dissociation dynamics to and from the protein array edges over tens of minutes (Fig. 2a–d dashed outlines, Supplementary Movies 3–6, illustrated experiments are in C18). These movies were recorded ~40 min (Fig. 2a, Supplementary Movies 3), ~120 min (Fig. 2b, Supplementary Movies 4), ~40 min (Fig. 2c, Supplementary Movies 5) and ~15 min (Fig. 2d, Supplementary Movies 6) after continuous bilayer formation. Importantly, the AqpZ 2D-arrays continued to change shape with local growth and contraction but without global changes in array size (Fig. 2e, f). Thus, the association-dissociation events analyzed here were recorded under equilibrium conditions. We distinguished the association-dissociation events as either one-bond or two-bond events, defined by the number of neighbor interactions of a molecule in the AqpZ array (Fig. 2b–d). We analyzed only complete events, defined as the process in which a diffusing AqpZ associated to and then dissociated from an array, thus defining bound-state dwell times (Supplementary Fig. 3). We captured a large number of complete one-bond (Fig. 2b, asterisk 1) and two-bond (Fig. 2b, asterisk 2) events over extended experimental durations (Supplementary Movies 4–6). Association-dissociation events to and from three-bond locations were rare and were not analyzed.

HS-AFM imaging of one-bond and two-bond association-dissociation events revealed exponential dwell-time distributions with distinct time constants (τ_1_ and τ_2_). We applied two strategies to analyze the state dwell times: First, we treated the one-bond (Fig. 2g, left) and two-bond (Fig. 2g, center) events separately, based on the protein array images, where the molecular environment was entirely unchanged before and after association-dissociation. Accordingly, the one-bond dwell-time distribution was well described with a single exponential decay time constant τ_1_ = 0.81 s (*n* = 246), and the two-bond dwell-time distribution decay with a time constant τ_2_ = 7.4 s (*n* = 549). This strategy established that the two interaction morphologies, one-bond *vs* two-bond, had significantly different dwell times,  and assigned the fast time constant to one-bond and the slow time constant to two-bond events. Second, all events were pooled, and the resulting dwell-time distribution was fit with a double exponential (Fig. 2g, right):

1 $$P\left(B\right)={c}_{1}{{{{{{\rm{e}}}}}}}^{-\frac{t}{{\tau }_{1}}}+(1-{c}_{1}){{{{{{\rm{e}}}}}}}^{-\frac{t}{{\tau }_{2}}}$$

where *P(B)* is the normalized cumulative probability of all events (*n* = 1096). τ_1(C18)_ = 0.77 s and τ_2(C18)_ = 8.2 s were the fast and slow time constants of the exponential decay, and c_1(C18)_ = 0.55 and c_2(C18)_ = 0.45 (c_2_ = 1-c_1_) represented the relative abundance of the fast and slow events, respectively. The fast and slow time constants, τ_1_ and τ_2_, agreed well with the time constants individually determined for the one-bond and two-bond events based on imaging knowledge, but the ensemble fitting quality was better. Therefore, we used ensemble fitting to analyze each individual array in all experiments to assess detailed statistics with error estimates between experimental observations (Table 1).

**Table 1:** Statistics of AqpZ_4_ association/dissociation kinetics and diffusion in C14, C16, C18 and C20 lipid bilayers

	τ1 (s)	τ2 (s)	*D*_*U*_ (µm^2^/s)	*C*_*U*_ *(*µm^−2^)
C14	0.5 ± 0.14 (0.7)	13 ± 3.3 (16 ± 4.9)	0.42 ± 0.051	110 ± 39
C16	0.7 ± 0.10 (0.7)	9 ± 3.2 (8 ± 3.4)	0.64 ± 0.039	30 ± 18
C18	0.9 ± 0.10 (0.7)	9 ± 1.1 (8 ± 1.7)	0.59 ± 0.038	30 ± 10
C20	0.6 ± 0.10 (0.7)	15 ± 1.2 (17 ± 1.8)	0.48 ± 0.031	160 ± 93

The time constants τ_1_ and τ_2_ were determined using Eq. (1). Brackets: Alternative fitting strategy: Fixing τ_1_ to 0.7 s and optimizing τ_2_. The 2D diffusion coefficients *D*_2D_ and 2D concentration *C*_U_ were determined using HS-AFM-HS. All statistics (mean ± s.e.) were determined from three biological replica in each condition.

Membrane elastic theory proposes that hydrophobic mismatch between the bilayer core and the protein transmembrane domain (TMD) controls membrane protein interactions^15,16,36,37^. To test this, we repeated the above experiments and analyses in membranes constituted of synthetic purified lipids with different thicknesses. In addition to DOPC (C18), we also used 1,2- dimyristoleoyl-sn-glycero-3-phosphocholine (C14), 1,2-dipalmitoleoyl-sn-glycero-3-phosphocholine (C16), and 1,2-dieicosenoyl-sn-glycero-3-phosphocholine (C20) in the lipid addition step (Fig. 1e, step 2). These lipids have the same degree of saturation but different hydrocarbon tail lengths (Supplementary Fig. 4), with a ~1.5 Å increase in bilayer thickness for each additional carbon atom, ranging from ~24 Å for the C14 bilayer to ~34 Å for the C20 bilayer^38^. The phase transition temperatures for all these lipids were far below room temperature, and thus all bilayers were in the fluid phase in our experiments. The dynamics of AqpZ, which has a hydrophobic thickness of ~28.6 Å matching a hypothetical C17 bilayer (Supplementary Fig. 5, “Methods”), were analyzed in C14, C16, C18, and C20 membrane environments, revealing that indeed, the membrane thickness had an influence on the membrane-mediated protein interactions (Table 1, columns 1 and 2, Fig. 2h).

Next, we exploited HS-AFM height spectroscopy (HS-AFM-HS, see “Methods”)^35^ to characterize the diffusion of unbound molecules that are not resolved in images (Fig. 2i). HS-AFM-HS captured the height fluctuations induced by molecules diffusing under the tip with µs temporal resolution, far away, ~100 nm from the border of an AqpZ 2D-array, in the bilayer membrane. Analysis of height-time traces (Fig. 2i, middle) revealed the 2D-diffusion coefficient, *D*_*U*_ (µm^2^ s^−1^), of the freely diffusing AqpZ molecules using ${\tau }_{D}={w}^{2}/4{D}_{U}$ and the 2D-concentration of unbound AqpZ, *C*_*U*_ (µm^−2^) through ${C}_{U}={t}_{(z > {H}_{{{{{{\rm{T}}}}}}})}/{t}_{({{{{{{\rm{total}}}}}}})}\cdot 1/{A}_{{{{{{{\rm{AqpZ}}}}}}}}$, where *τ*_*D*_ is the dwell-time of the diffusion events, and *w* is the detection area estimated from the area of an AqpZ, *A*_AqpZ_, convoluted with the tip radius (~1 nm)^35^. *t*_(*z>H*T)_ is the total time the tip detects diffusion events at a height *z* above *H*_T_ = 5σ of the height value distribution, during *t*_(total)_, the total measurement time^35,39^. The diffusion events had a height between 1 nm and 1.5 nm above the membrane, i.e. baseline (Fig. 2i, middle), agreeing well with the height of the diffusive area (Fig. 1g, i, labeled D), and with the protrusion height of array-bound cytoplasmic, ~1 nm, and extracellular, ~1.5 nm, face exposing AqpZ (Fig. 2b–d). We found that the diffusion coefficient *D*_*U*_ of AqpZ also varied with the bilayer thickness (Table 1, column 3, Fig. 2i).

Both the state dwell times and the diffusion speed were altered by changes in lipid bilayer thickness (Table 1). Notably, AqpZ in bilayers of intermediate thickness, closest to the hydrophobic thickness of the protein, displayed longer *τ*_*1*_ and shorter *τ*_*2*_, as well as faster *D*_*U*_. Since *τ*_*1*_ from the fitting is slightly shorter than the imaging rate, 1 frame/s, we performed two additional tests. First, we tested the fitting for *τ*_*2*_ while keeping *τ*_*1*_ fixed at *τ*_*1*_ = 0.7 s (the average *τ*_*1*_ across all lipids). This constrained fitting strategy confirmed the state dwell-time trend, where *τ*_*2*_ was prolonged in membranes with increased hydrophobic mismatch (Table 1, brackets in columns 1 and 2). Second, we imaged AqpZ 2D-arrays in DOPC at 4 frames/s (Supplementary Movie 7; at proportionally smaller scan size but identical pixel sampling as the experiments at 1 frame/s), and estimated τ_1(C18)_ = 0.54 ± 0.09 s and τ_2(C18)_ = 8.0 ± 1.1 s, thereby supporting the sub-second τ_1_ derived from the 1 frame/s data (Table 1, column 1).

### A kinetic model of membrane-mediated protein interactions

A first analysis of the interactions can be made by considering the equilibrium between freely diffusing molecules (*U*) dissolved in the lipid membrane, and the bound molecules (*B*) that form the arrays. This equilibrium is associated with the reaction:

2 $$U\mathop{\leftrightharpoons }\limits^{{K}_{{{{{{\rm{eq}}}}}}}^{U-B}}B,$$

where ${K}_{{eq}}^{U-B}={C}_{B}/{C}_{U}$ is the equilibrium constant for this reaction, and *C*_*B*_ is the concentration of bound and *C*_*U*_ the concentration of unbound molecules. Thus, the association energy (*ΔG*^*0*^_asso_) is

3 $${\Delta G}_{{{{{{{\rm{asso}}}}}}}}^{0}={-k}_{B}T{{{{{\rm{ln}}}}}}\left(\frac{{C}_{B}}{{C}_{U}}\right)$$

We know *C*_*B*_ precisely from cryo-EM (Fig. 1c, Supplementary Fig. 2) and HS-AFM (Fig. 1d) imaging. In the arrays one AqpZ tetramer occupies 45.125 nm^2^, and thus *C*_*B*_ is 22,161 µm^−2^. We measured, using HS-AFM-HS, the concentration of freely diffusing molecules, *C*_*U*_, in all bilayers (Fig. 2i, Table 1, column 4). From these two measurements, *ΔG*^*0*^_asso_ is calculated according to Eq. (3) (Table 2, column 1). The intuition of Eq. (3) is to relate the association energy (*ΔG*^*0*^_asso_) between unbound and bound molecules to the equilibrium concentrations of the two species.

**Table 2:** Energies of membrane-mediated AqpZ interactions in C14, C16, C18 and C20 lipids

	*ΔG*^*0*^*P-P*(asso) (*k*_B_T)	*ΔG*^*0*^asso (*k*_B_T)	*ΔG*^*0*^*P-P*(diff) (*k*_B_T)	*ΔG*^*0*^diff (*k*_B_T)
C14	−6.9	−5.4 ± 0.41	−2.3	−3.2 ± 0.41
C16	−6.9	−6.8 ± 0.61	−2.3	−2.5 ± 0.27
C18	−6.9	−6.6 ± 0.34	−2.3	−2.3 ± 0.13
C20	−6.9	−5.1 ± 0.56	−2.3	−3.2 ± 0.25

The energies were determined based on the measured state dwell times from HS-AFM imaging and HS-AFM-HS as described in the text.

To get further insights into the single-molecule behavior in different B states, we consider a simple kinetic three-state model (Fig. 2j). In this model, the unbound AqpZ (U, red) could bind to an array (A, gray) in one of two possible modes: either in a one-bond (1B, green) or in a two-bond (2B, blue) site. The association-dissociation events, directly observed by HS-AFM at the single-molecule level, were found to follow first order kinetics from both 1B and 2B states. Accordingly, the dissociation of a molecule making one contact with the array, state 1B, is fast, i.e., the bond lifetime is short. Making an additional contact to the array by filling a gap in a corner, state 2B, stabilizes the bound state and its dissociation is slow, i.e., the bond lifetime is long. Thus, the energy difference between states 1B and 2B, the energy gain of the second bond formation (*ΔG*^*0*^_diff_), can be estimated as^40^

4 $$\Delta {G}_{{{{{{{\rm{diff}}}}}}}}^{0}=-{k}_{B}T{{{{{\rm{ln}}}}}}\left(\frac{{\tau }_{2}}{{\tau }_{1}}\right)$$

The intuition of Eq. (4) is that i) the two bound states, 1B and 2B, interconvert to the same unbound state through the same transition state, and ii) according to simple rate theory, the logarithm of the unbinding rate is proportional to the energy barrier height. Therefore, the logarithm of the ratio of the dwell times is related to the energy difference of the two bound states.

Based on the measured state dwell times (Table 1) and the kinetic model (Eqs. (3) and (4)), the association *ΔG*^*0*^_*asso*_ of an unbound AqpZ to others is favored (~−6.7 *k*_B_T) in C16 and C18 membranes matching the protein hydrophobic thickness, while it is less favored (~−5.3 *k*_B_T) in C14 and C20 membranes (Fig. 2k, top, Table 2, column 2). In direct analogy with an elastic potential energy, *ΔG*^*0*^_asso_ can be plotted as a function of the square value of the hydrophobic mismatch, *u*_*0*_^2^, and extrapolated to zero mismatch at an energy of *ΔG*^*0*^_*P-P(*asso*)*_ of −6.9 *k*_B_T (Fig. 2k, bottom), suggesting a favorable membrane-independent protein–protein interaction energy (Table 2, column 1). In contrast, *ΔG*^*0*^_diff_ is lower (~−3.2 *k*_B_T) in membranes of the shorter and longer lipids, C14 and C20, than in C16 and C18 membranes (~−2.4 *k*_B_T) that match the hydrophobic core of the protein (Fig. 2l, top, Table 2, column 4). Again, the energies can be characterized by fitting a quadratic curve (Fig. 2l, top), and are therefore plotted as a function of the square of the hydrophobic mismatch, *u*_*0*_^2^ (Fig. 2l, bottom), and the intercept provides an estimate of the membrane mismatch-independent protein–protein interaction energy *ΔG*^*0*^_*P-P(*diff)_ = −2.3 *k*_B_T (Table 2, column 3). The difference of the membrane-independent protein–protein interaction energies calculated from these two independent approaches, *ΔG*^*0*^_*P-P(*asso*)*_ = −6.9 *k*_B_T, characterizing the overall association of a free molecule to an array, and *ΔG*^*0*^_*P-P(*diff)_ = −2.3 *k*_B_T, characterizing the bond strengthening by one additional protein partner, is well explained by the fact that an average array-bound AqpZ molecule has ~3 interactions with neighbors.

Comparing the protomer mobility in different bilayer thicknesses, the measured diffusion coefficient was found to decrease with increasing hydrophobic mismatch (Fig. 2m, Table 1, column 3). This finding agrees with reported deviations from Saffman–Delbrück diffusion^39^ due to an increase in the effective membrane viscosity that scales linearly with membrane mismatch^41,42^. Extrapolation of these measurements indicate that AqpZ would attain a maximal value of *D*_*U*_ = 0.7 µm^2^/s in a perfectly matching bilayer in our experimental system (Fig. 2m, bottom). We consider that the underlying mica may affect diffusion through interaction with the proteins and/or modulation of the bilayer physical properties, but note that the atomically flat mica does not provide diffusion obstacles and a diffusion coefficient of 0.7 µm^2^/s is a rather typical value for a membrane protein of the size of AqpZ.

In summary, the interaction energies emerging from hydrophobic mismatch account for ~1.5 *k*_B_T (Table 2), complemented by a larger direct protein–protein mismatch-independent energy. Importantly, membrane-mediated membrane protein interactions are long-range – membrane proteins sense each other through the membrane at distances far beyond the range where electrostatic and Van der Waals interactions become important. Thus, membrane-mediated membrane protein interactions represent a key driving force in the organization of membrane proteins.

### Membrane-mediated interactions are long-range and geometry-sensitive

**Fig. 3:**
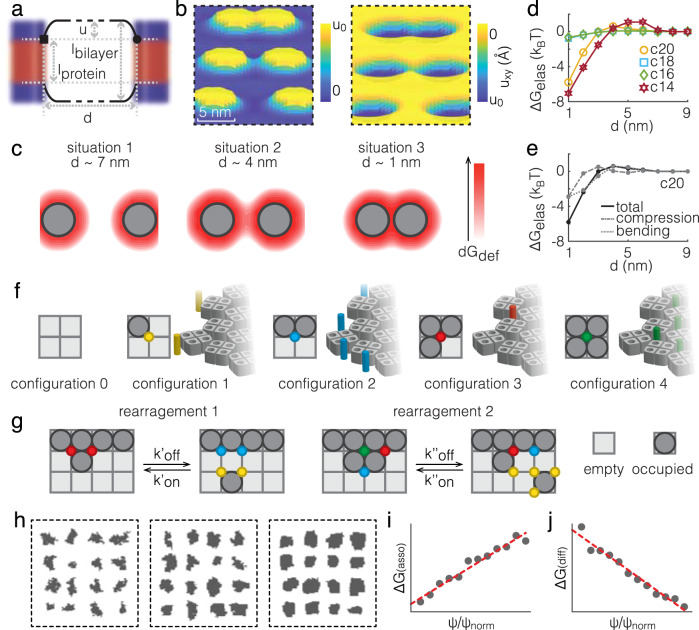
Membrane deformation through membrane protein hydrophobic mismatch provides a theoretical understanding of the experimental results. **a**–**e** Hydrophobic mismatch as an energy source for membrane-mediated membrane protein interactions (Supplementary Fig. 7, Supplementary Note 1). **a** Schematic of the inclusion-induced membrane deformation with l_protein_, protein hydrophobic thickness, l_bilayer_, bilayer hydrophobic thickness, u, hydrophobic mismatch, and d, edge-to-edge distance between proteins (hydrophobic (red) and hydrophilic (blue) protein surfaces). **b** Perspective schematic representation of 2D membrane profiles (one leaflet) when two cylindrical proteins approach. The space occupied by proteins is not considered part of the deformation field, *u*_*xy*_, and filled with *u*_0_ for illustration: positive, (e.g., C14, left) and negative (e.g., C20, right) hydrophobic mismatch. Bottom to top: d ~7 nm, ~4 nm, and ~1 nm. **c** Schematic representation of the 2D deformation energy density, dG_def_, for d ~7 nm, ~4 nm and ~1 nm. **d** 2D elastic mismatch-dependent energy potential as a function of d for the four investigated bilayers. **e** Compression and bending components of the total 2D elastic mismatch-dependent energy potential, repulsive from ~7 nm to ~3.5 nm separation, and attractive at separation shorter ~3.5 nm. **f**, **g** Changes of 2D membrane configurations in association-dissociation events (Supplementary Fig. 9): **(f)** The five basic local-configurations in microscopic array assembly. **g** Representative rearrangements of local configurations associated with one-bond (rearrangement 1) and two-bond (rearrangement 2) interactions. **h**–**j** Membrane protein automata (Supplementary Fig. 10, Supplementary Note 2, and Supplementary Movie 8): (**h**) Simulated clusters in membranes of no (left), intermediate (middle), and large (right) hydrophobic mismatch. **i** Association energy (ΔG_asso_), and (**j**) energy difference between states 1B and 2B (Δ*G*_diff_), as functions of the deformation energy scale factor ψ/ψ_norm_, representing the hydrophobic mismatch square (u_0_^2^), with ψ_norm_ = {1.00 2.06 3.22 4.10} (see “Methods”).

Past experimental and theoretical work on interactions between integral membrane proteins and the lipid bilayers in which they are imbedded has provided physical models that explicitly estimate the membrane deformation energetics and the impact of hydrophobic mismatch as a function of distance (*d*) between membrane proteins (Fig. 3a). Each membrane protein deforms the membrane at its circumference to match the hydrophobic core of the membrane with its hydrophobic membrane exposed residues, so that unfavorable interactions between lipid hydrocarbon tails and hydrophilic residues on the inner and outer brim of the protein are minimized^15^. The membrane deformation is approximated as a 2D continuous elastic field, *u*_*xy*_, representing the deviation of the lipid head-group from its unperturbed height^16^. The hydrophobic mismatch of one leaflet *u* is *u*_*0*_ at the protein-lipid interface and vanishes to zero as the membrane becomes unperturbed. The deformation energy, *G*_*def*_, in this setting results from membrane compression and bending (Supplementary Fig. 6, Supplementary Note 1), and both have a form analogous to Hooke’s law. Thus all components contribute to elastic energy. The expression of *G*_def_ is

5 $${G}_{{{{{{{\rm{def}}}}}}}}=\frac{1}{2}\int \int \left[{K}_{A}{\left(\frac{{u}_{{xy}}}{l}\right)}^{2}+{\kappa }_{b}{\left({\nabla }^{2}{u}_{{xy}}\right)}^{2}\right]{{{{{{\rm{d}}}}}}x{{{{{\rm{d}}}}}}y},$$

where *K*_*A*_ is the bilayer stretch modulus, *l* the thickness, *κ*_*b*_ the bending modulus, and ${\nabla }^{2}=\frac{{\partial }^{2}}{\partial {x}^{2}}+\frac{{\partial }^{2}}{\partial {y}^{2}}$ the Laplace operator. Since the 2D deformation energy associated with multiple proteins depends on the complex geometries of the membrane and protein configuration^43^, i.e. the shapes of the protein cross-sections as well as the distances and orientations relative to each other, etc., we first illustrate the intuition of *G*_def_ generated by two cylindrical proteins as a simple case (Fig. 3a–e).

We solved the 2D continuous elastic field *u*_*xy*_ induced by two cylindrical proteins of identical membrane mismatch at different edge-to-edge distances *d* through numerical simulation (Supplementary Fig. 7)^44^. If the protein centers are positioned along the *x*-axis, and the closest protein-lipid interfaces are at (*x*_*1*_,*0*) (Fig. 3a, black square) and (*x*_*2*_,*0*) (Fig. 3a, black circle), then *u*_*x1,0*_ = *u*_*x2,0*_ = *u*_*0*_ and *d* = *|x*_*2*_ *– x*_*1*_| (Supplementary Note 1). The membranes adopt different profiles between the proteins as the two molecules approach (Fig. 3b). The membrane perturbation around each molecule relates to deformation energy, *G*_def_, (Fig. 3c), which is the spatial integral of the deformation energy density *dG*_def_. Thus, the change of the deformation energy in the approach of two molecules gives the elastic potential, *ΔG*_elas_(*d*) (Fig. 3d), as

6 $$\Delta {G}_{{{{{{{\rm{elas}}}}}}}}(d)={G}_{{{{{{\rm{def}}}}}}}(d)-{G}_{{{{{{{\rm{def}}}}}}}}(+{{\infty }})$$

Due to the physical properties of the membrane, the elastic potential is ‘felt’ by membrane proteins that are as far as ~7.5 nm apart, and scales with the hydrophobic mismatch (Fig. 3c, situation 1, Fig. 3d). At *d* ~7.5 nm to ~3.5 nm, as the deformed membrane fields overlap, the potential is repulsive, especially in the case of large hydrophobic mismatch. This is primarily due to the membrane bending component that has to accommodate a saddle-shaped membrane topography at such intermediate distances (Fig. 3c, situation 2, Fig. 3d, e). Decreased membrane bending and compression at *d* < ~2 nm produce strongly attractive potentials at short distances (Fig. 3c, situation 3, Fig. 3d, e). These results are consistent with previous theoretical studies treating the approach of two ideal cylindrical inclusions in a membrane^16,45,46^.

The expected membrane deformation was observed in the space between 4 AqpZ tetramers. In this region the membrane formed a saddle point with a height variation of ~1 Å, though this measurement must be taken with caution because only very sharp tips can potentially probe this region between the proteins (Supplementary Fig. 8). To relate membrane elastic theory to the experimental observations described in Fig. 2, we developed a discretized framework that evaluates the changes of the membrane environment associated with membrane protein assembly configuration changes. In this approach, we approximate the membrane as a lattice, each point of which is either occupied by a molecule or empty (Fig. 3f). A 2 × 2 region of the membrane lattice has five distinct configurations depending on the number of molecules that occupy the positions and may be used to assign deformation energies to the various configurations (see “Methods”). We denote *ψ*_*i*_ as the deformation energy of local-configuration *i*. Thus, we can write the membrane-dependent energy change from a membrane configuration rearrangement, e.g., due to a protein association or dissociation event, *Δψ*, as

7 $$\Delta \psi={\delta n}_{1}{\psi }_{1}+{\delta n}_{2}{\psi }_{2}+{\delta n}_{3}{\psi }_{3}+{\delta n}_{4}{\psi }_{4},$$

where *δn*_*i*_ is the change, gain or loss, of local-configuration *i* in the rearrangement. For example, for the one-bond and two-bond association events shown in Fig. 3g, {*δn*_1_ *δn*_2_ *δn*_3_ *δn*_4_} equals {−2 −2 2 0} (rearrangement 1) and {−4 0 0 1} (rearrangement 2), respectively, allowing us to compute the membrane morphological changes (Fig. 3g, Supplementary Fig. 9). Hence, we developed a simulation, termed membrane protein automata (Supplementary Note 2, see “Methods”), with which we simulate distinct membrane protein organizations through varying the energy term of the direct protein–protein interaction *E*_*P-P*_, the energy of the relative local-configurations, *ψ*_*1*_, *ψ*_*2*_, *ψ*_*3*_ and *ψ*_*4*_, and the concentration of the freely diffusing molecules *C*_*U*_ (Supplementary Fig. 10).

To determine {*ψ*_*1*_ *ψ*_*2*_ *ψ*_*3*_ *ψ*_*4*_}, we solved through numerical simulations the 2D continuous elastic field *u*_*xy*_ of local-configurations 1 to 4 (Supplementary Note 1, Supplementary Fig. 7)^44^. Using Eq. (7) to evaluate the rearrangements in Fig. 3g shows that the rearrangement 2 is much more favorable than rearrangement 1, since *Δψ*_re2_-*Δψ*_re1_ < 0. Thus, the rectangular shape of the observed arrays with neat borders and without protruding molecules is favored over more fuzzy protein assemblies (Figs. 1i, 2a–d, Supplementary Fig. 9).

We performed extensive simulations (Fig. 3h, Supplementary Fig. 10, Supplementary Note 2), linearly scaling {*ψ*_*1*_ *ψ*_*2*_ *ψ*_*3*_ *ψ*_*4*_} to simulate the effect of hydrophobic mismatch square (*u*_0_^2^). The membrane protein automaton generated protein arrays that displayed similar morphology as in the experiment (Fig. 3h). Analysis of the simulated association/dissociation events revealed similar membrane-dependent energetic trends as in experiments, where *ΔG*_asso_ scales positively and *ΔG*_diff_ scales negatively with increasing membrane mismatch square (Fig. 3i, j; compared to Fig. 2k, l).

### Lipid thickness matching the protomer interface destabilizes oligomers

**Fig. 4:**
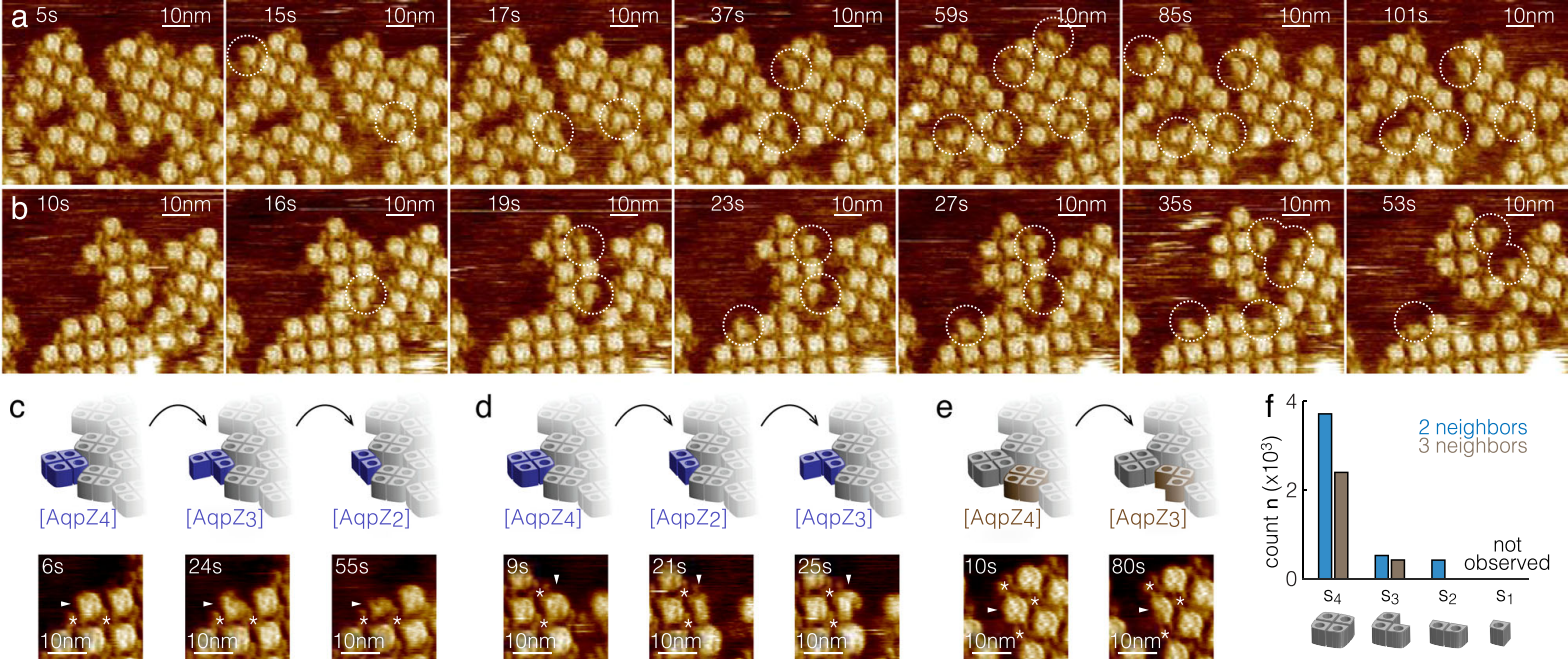
AqpZ W14A protomer association and dissociation dynamics in a lipid bilayer that matches the hydrophobic thickness of the AqpZ protomer-protomer interface. **a**, **b** HS-AFM movie frames (Supplementary Movies 9,10) of non-canonical AqpZ oligomers, AqpZ_2_ and AqpZ_3_ in a C20 membrane (image parameter: 0.33 nm/pixel). Dashed circles highlight AqpZ_2_ and AqpZ_3_. **c**–**e** Oligomer transitions: (**c**) AqpZ_4_$\to$AqpZ_3_$\to$AqpZ_2_, **(d**) AqpZ_4_$\to$AqpZ_2_$\to$AqpZ_3_, and (**e**) AqpZ_4_$\to$AqpZ_3_ (arrowheads: molecule of interest; asterisks: neighbor molecules). Images are averages over 5 consecutive frames (if applicable) with time stamps corresponding to the first frame of state occurrence. **f** Occurrence probabilities of AqpZ W14A oligomeric states at the array edge.

Aquaporins are stable tetramers^47^, which precluded the analysis of the oligomerization mechanism. Therefore, we performed these experiments with a W14A mutant, a single residue mutation at the protomer interface. We reasoned that the exchange of the bulky tryptophan with the small alanine would allow penetration of membrane lipids into the interstices between the protomers of tetrameric AqpZ, AqpZ_4_, potentially destabilizing the protomer interaction (Supplementary Fig. 11)^30,48^. Thus, we expected to observe non-tetrameric AqpZ-W14A, which we denote AqpZ_1_ (monomers), AqpZ_2_ (dimers), and AqpZ_3_ (trimers). We screened the edges of the AqpZ arrays for these species in C14, C16, and C18 membranes, but without success. Interestingly, we reproducibly detected non-tetrameric AqpZ in high-resolution HS-AFM images in C20 lipids, where ~10% of the array-bound AqpZ had an oligomeric state that deviated from the tetrameric form (Fig. 4a, b, outlines, Supplementary Movies 9, 10). As a comparison, non-tetrameric oligomers were much rarer for WT AqpZ in C20 lipids, <2%, which suggests that the W14A mutation accounts for at least 2 *k*_B_T in the AqpZ oligomerization (Supplementary Fig. 12). In order to analyze occurrence probabilities and derive energetics of these states, the number of interactions with neighboring molecules needed to be considered: Due to protomer stabilization with the array molecules, an AqpZ_n_ with two neighbors can only dissociate into AqpZ_3_ or AqpZ_2_ (Fig. 4c, d), while an AqpZ_n_ with three neighbors can only become AqpZ_3_ (Fig. 4e). Transition to AqpZ_1_ would be possible if the array-bound AqpZ had only one neighbor, but we failed to capture such events, likely because of the very small size of AqpZ_1_ combined with the very short τ_1_ of the one-bond interaction (see Table 1). We estimated the energy differences between oligomeric states *s*_*1*_ and *s*_*2*_ from the numbers of observations, *N*_*s1*_ and *N*_*s2*_ with neighbors *n*, in the HS-AFM imaging period, as:

9 $$\Delta {G}_{s2,\,n}^{0}-\Delta {G}_{s1,n}^{0}=-{k}_{B}T{{{{{\rm{ln}}}}}}\left(\frac{{N}_{s2,n}}{{N}_{s1,n}}\right)$$

We found that regardless of the neighbor number, AqpZ_2_ and AqpZ_3_ had similar occurrence probabilities, and thus energy difference compared to AqpZ_4_, ~2 *k*_B_T (Fig. 4f, Table 3), similar in magnitude as the W14A mutation. These estimated energy differences are hardly comparable to those between freely diffusing oligomers because i) HS-AFM experiment requires oligomers having >1 contact with the array for detection, hence underestimating the real numbers, and ii) the constrained molecular environment at the array edges reduces the degree of freedom of movements.

**Table 3:** Energies of AqpZ-W14A oligomerization in C20 lipid

Δ*G*^*0*^_(C20)_ (*k*_B_T)	1 neighbor	2 neighbors	3 neighbors
AqpZ_1_-AqpZ_2_	N/A	N/A	N/A
AqpZ_2_-AqpZ_3_	N/A	0.0 ± 0.61	N/A
AqpZ_2_-AqpZ_4_	N/A	−2 ± 1.1	N/A
AqpZ_3_-AqpZ_4_	N/A	−2 ± 1.1	−2.1 ± 0.51

The energies were determined as described in the text. AqpZ_1_ is not accessible in both the 2-neighbor and 3-neighbor situations. AqpZ_2_ is not accessible in the 3-neighbor situation. Energies in the 1-neighbor situation were not analyzed due to poor statistics.

To understand why low-order oligomers occurred in C20 lipids and not in thinner membranes (C18, C16, and C14), we assessed the hydrophobic thickness of the AqpZ protomer-protomer interface and found that it was very different from the hydrophobic thickness that the tetramer exposes to the membrane. Indeed, we assessed a hydrophobic thickness of ~28.6 Å on the membrane exposed surface but estimated a hydrophobic thickness of ~33.0 Å between protomers (Supplementary Fig. 5c). This much thicker hydrophobic interface matches roughly the thickness of the C20 lipids. Thus, bilayers with a hydrophobic core that matches protomer interfaces lower the energy difference between the oligomeric state and individual protomers, favoring dissociation.

Strikingly, owing to the experimental design with free membrane outside the arrays and the time-resolved imaging of HS-AFM, not only overall statistics of the occurrence of AqpZ_n_ could be assessed, but also real-time transitions AqpZ_4_ → AqpZ_3_ → AqpZ_2_ (Fig. 4c) AqpZ_4_ → AqpZ_2_ → AqpZ_3_ (Fig. 4d) and AqpZ_4_ → AqpZ_3_ (Fig. 4e) could be observed. Given that these transitions are very slow, tens of seconds for the dissociation transitions (Fig. 4c, *t* = 6 s, *t* = 24 s, *t* = 55 s, Fig. 4d, *t* = 9 s, *t* = 21 s and Fig. 4e, t = 10 s, *t* = 80 s), we estimate that the energy barrier between AqpZ_4_ and AqpZ_3,2_ is very high, ~24 *k*_B_T in the experimental conditions (see “Discussion”).

## Discussion

**Fig. 5:**
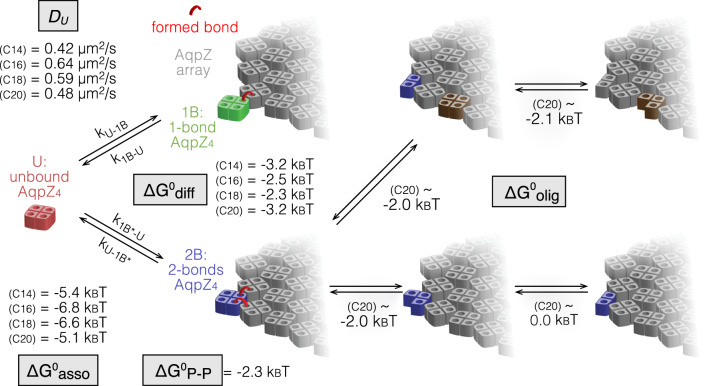
AqpZ oligomerization and assembly. The AqpZ-W14A oligomerization energetics was estimated based on observation statistics of non-tetrameric complexes. The protomer interaction ΔG^0^_olig_ is weakest (~−2 *k*_B_*T*) in C20 lipids that match the hydrophobic thickness of the protomer interface. AqpZ diffusion D_U_ is slowed in lipids with larger hydrophobic mismatch (thicker and thinner). Membrane-mediated membrane protein interaction Δ*G*^0^_asso_ is most favorable (~−6.5 *k*_B_*T*) in lipids with thickness close to the hydrophobic thickness of the protein. Bond formation with two array-bound proteins, filling gaps in the 2D-plane, provides a maximum energy gain Δ*G*^0^_diff_ in lipids with strong mismatch (~−3 *k*_B_*T*). The latter driving the assembly towards the formation of membrane protein arrays. The direct (not membrane-mediated) protein–protein interaction ΔG^0^_P-P_ (~−2 *k*_B_*T*) stabilizes these interactions at very short distances.

Here, we developed an approach to investigate membrane-mediated protein interactions in a controlled manner and at single-molecule resolution (Fig. 5). Membrane protein patches that contained very little lipid (LPR 0.1) and covered only a small portion (~5%) of the sample surface were supplied with lipids of defined hydrophobic thickness to form a continuous fluid lipid bilayer in which the membrane proteins diffused and interacted. Taking HS-AFM movies of this system, thousands of membrane protein association/dissociation events were recorded in C14, C16, C18, and C20 PC bilayers, and their dwell times analyzed. Besides, HS-AFM-HS was applied for the analysis of diffusing molecules, including their 2D concentrations and diffusion coefficients. Based on these measures, together with a mechanical model of the lipid bilayer, we found that the interaction energies scaled with the hydrophobic mismatch between protein and the bilayers. In our model system, the protein–protein association was more favorable in lipids matching the protein’s hydrophobic thickness, but the engagement with multiple neighbors in protein array formation was more favorable in bilayers with large mismatch. We note that the tested lipids have similar *K*_*A*_ values^49,50^. In principle *κ*_*b*_ is proportional to *K*_*A*_ and the bilayer thickness, *κ*_*b*_ ~*K*_*A*_/l^2^, thus *κ*_*b*_ is expected to be somewhat larger for thinner bilayers. However, we found that C14 and C20 lipids had very similar energetics and suggest therefore that the apparent proportionality of *κ*_*b*_ with thickness is weak.

Based on 1D membrane deformation graphs, one might be led to think that large hydrophobic mismatch must favor membrane protein association (Fig. 3a). However, in the 2D membrane, protein association in mismatched membranes leads to complex local membrane deformations, e.g., saddle-points (Fig. 3b, c, Supplementary Fig. 8), that – as our experiments show – dominate the interactions and are overall unfavorable. Furthermore, the extrapolation of the mismatch potential energy dependence of these associations allowed us to determine the energy of the direct protein–protein interaction. All interaction energies we found are in the single digit −*k*_B_T range (Fig. 2, Table 2). We reason that such single digit -*k*_B_T range energy differences provide effective biases without locking the molecules in specific states, and thus leave membrane proteins amenable to rearrangements. We note however that our calculations consider an average hydrophobic thickness for the protein, but membrane protein surfaces have local variation of the hydrophobic thickness, and thus the membrane-mediated protein interaction strength is also expected to vary locally. While the state energy differences are relatively low, we note that the dwell times, *τ*, of the interactions were long, especially for the molecules engaging in multiple bonds, ~10 s (and for the protomer interaction, tens of second), suggesting that the bound and unbound states are separated by high energy barriers. We can estimate the barrier height *ΔE*_barrier_ from the measured dwell times using 1/*τ* = *A*exp(−*ΔE*_barrier_/*k*_B_T), where *A* is an unknown pre-factor. Using *A* ~10^9^–10^10^ s^−1^, estimated for a bond rupture in a viscous medium^51^, this approximation predicts an *ΔE*_barrier_ ~23–25 *k*_B_T for interactions with *τ* ~10 s. Thus, the membrane protein tertiary structure and supra-molecular assembly are kinetically trapped by high energy barriers.

Using continuous elastic field modeling for the interpretation of the protein-induced membrane deformation required consideration of the membrane 2D geometry and the protein configuration. Solving the deformation fields relevant to the membrane protein arrays of hundreds of molecules would necessitate significant computational resources. To this end, we introduced a discretized framework, the membrane protein automata, to evaluate the morphological changes and the dynamics of membrane protein assemblies. The complex array association/dissociation events are considered as rearrangements of a finite number of local configurations, in which the deformation fields are readily solved. These automata can, with a fixed set of parameters obtained from numerical simulations of the local configurations, reproduce the dependence of protein array self-assembly and dynamics, as well as its sensitivity to hydrophobic mismatch. Thus, this framework serves as a simple and complementary approximation to the elastic continuous model. The automata produced protein assemblies that matched the experimentally observed protein arrays, suggesting that the understanding of molecular association/dissociation kinetics are sufficient to account for the equilibrium large-scale organization. Membrane protein arrays could potentially be analyzed from the perspective of the stability of the arrays, rather than from the perspective of the individual protein component, as has been done for rafts and nanodomains. The observed dynamics along the array edges would then resemble the raft formation, size fluctuations and raft merging processes^52^, and the array dynamics could be treated using an entropic analysis, though this might necessitate large-scale imaging comprising multiple arrays^53^.

Non-tetrameric AqpZ-W14A (W14A destabilizes the protomer interface) were found in C20 bilayers, suggesting that the membrane mechanical properties are also involved in stabilizing membrane protein oligomerization. In this regard, it is interesting to note that in eukaryotic cells, membrane proteins are synthesized and assembled into their native oligomeric state in the endoplasmic reticulum (ER), which has a particularly thin membrane compared to the plasma membrane^54^. Perhaps the ER membrane stabilizes oligomers due to enhanced hydrophobic mismatch, and this may influence post-translational modifications, sorting, and trafficking in the secretory pathway^55–57^. Dynamic imaging resolves the association/dissociation of monomers, indicative that monomeric aquaporin protomers are stable in membranes, shedding light onto the question how membrane proteins of complex quaternary structure may post-translationally oligomerize after release from the ribosome-translocon complex^58–60^.

Here, we show for the case of AqpZ that membrane organization can emerge from and is modulated by Brownian diffusion and a set of physical properties of the membrane constituents (Fig. 5). Further work is needed to test how other membrane proteins with different oligomeric states and shapes behave in such experiments. HS-AFM of unlabeled proteins, seeing not only single molecules of interest, but also their complex molecular environment, and revealing their dynamics, offers unique experimental possibilities to study membrane-mediated protein interactions.

## Methods

### Plasmid construction

Protein was expressed from a pET22-6His-TEV-Linker-AqpZ-W14A plasmid derived from a pTrc-10His-AqpZ plasmid^61^. The AqpZ gene was amplified by PCR from the pTrc plasmid and inserted in an empty pET22-6His-TEV plasmid by restriction-ligation cloning using the EcoRI and XhoI restriction sites^62^. The W14A mutation and a linker were introduced using megaprimer based mutagenesis^30,63^. The linker (sequence: SGSGSG) was inserted between the glycine of the TEV cleavage site and the methionine on the N-terminus of the AqpZ gene. Inserting this linker enabled His-tag cleavage by TEV protease presumably by bringing the TEV cleavage site into the aqueous environment^64^. All constructions were verified by sequencing.

### Protein expression

The pET22-6His-TEV-Linker-AqpZ-W14A plasmid was transformed into *E. coli* competent cell strain C41 ∆ompF ∆acrAB for protein overexpression^65^. Cells were grown on Luria broth (LB) plates supplemented with 100 μg/mL ampicillin at 37 °C. Cells from a single isolated colony were inoculated into LB media with 100 μg/mL ampicillin and incubated at 37 °C for 15 h. The overnight culture was diluted 100-fold into fresh LB broth and grown to an optical density at 600 nm (OD) between 1.2 and 1.5. AqpZ expression was induced by adding 1 mM isopropyl β-D-1-thio-galacto-pyranoside (IPTG), and cells were then incubated at 30 °C for 3 h at 180 rpm. Cells were harvested by centrifugation at 5000 *g* for 20 min. The cell pellet was washed with phosphate-buffered saline (PBS) and resuspended in 1/100 culture volume of a lysis buffer containing 50 mM Tris-HCl at pH 8.0, 100 mM NaCl, 10 mM MgCl_2_, 1 mM EDTA, 1 mM phenylmethylsulfonyl fluoride (PMSF) (Merck), 0.1 mg/mL DNase I (Roche), and 0.1 mg/mL Lysozyme (Merck). Cells were broken by three passages through a French press at 15,000 psi. Unbroken cells and debris were removed from the cell lysate by centrifugation at 5000 g for 20 min, and then membrane fragments were collected by centrifugation at 140,000 *g* for 45 min at 4 °C. The membrane pellet was then solubilized overnight at 4 °C in a solubilization buffer containing 50 mM Tris-HCl at pH 8.0, 100 mM NaCl, and 5% *n*-dodecyl-β-D-maltoside (DDM) (CliniSciences). The insoluble material was then removed by centrifugation at 210,000 g for 30 min.

### Protein purification

AqpZ was purified from the detergent-solubilized supernatant by nickel affinity chromatography using a 5 mL His-Trap HP column (GE Healthcare) attached to an ÄKTA system (GE Healthcare). The column was equilibrated with 5 column volumes (CV) of a washing buffer (W1) containing 100 mM Tris-HCl at pH 8.0, 150 mM NaCl, 0.1% DDM, and 100 mM imidazole. After proteins were loaded onto the column, the nonspecifically bound material was removed by washing with 5 CV of washing buffer W1. Elution was performed with a 5 CV gradient from 0 to 100% of an elution buffer containing 100 mM Tris-HCl at pH 8.0, 150 mM NaCl, 0.1% DDM, and 500 mM imidazole. Fractions containing AqpZ were pooled and loaded into dialysis tubing (SnakeSkin Dialysis Tubing 3.5 kDa, Thermo scientific), and dialyzed against 100 volumes of a dialysis buffer containing 100 mM Tris-HCl at pH 8.0 and 150 mM NaCl for 3 h at 4 °C. The dialyzed proteins were concentrated on a 1 mL His-Trap HP column. The column was equilibrated with 10 CV of a washing buffer (W2; without imidazole) containing 100 mM Tris-HCl at pH 8.0, 150 mM NaCl and 0.1% DDM. Proteins were loaded on the column and washed with 5 CV of washing buffer W2, followed by an elution step with 100 % elution buffer. Fractions containing the highest AqpZ concentration were pooled and dialyzed as above. The 6-His-tag was then removed by digestion with TEV protease. Protease was added to the purified AqpZ at a ratio of 1:5 (w/w) and the buffer was adjusted to contain 100 mM Tris-HCl at pH 8.0, 150 mM NaCl, 0.5 mM EDTA, 1 mM DTT, 20 % glycerol, and 0.1 % DDM. Digestion was allowed to continue overnight at room temperature. The cleaved AqpZ was then separated from the TEV protease by nickel affinity using a 1 mL His-Trap HP column. The column was equilibrated with 10 CV of washing buffer W2. After sample loading, the His-tag free AqpZ was recovered by washing the column with 5 CV of washing buffer W2. The fractions containing the protein were pooled and stored with 20% of glycerol at −80 °C.

### Aquaporin-Z W14A reconstitution and physisorption

Purified AqpZ W14A was solubilized in a buffer containing 100 mM Tris-HCl at pH 7.6, 150 mM NaCl, DDM (>3 critical micelle concentration, CMC), and 20% glycerol (protein buffer). The lipid mixture (1,2-dioleoyl-sn-glycero-3-phosphoethanolamine (DOPE), 1,2-dioleoyl-sn-glycero-3-phospho-L-serine (DOPS), 1,2-dioleoyl-sn-glycero-3-phosphocholine (DOPC), DOPC:DOPS:DOPE = 8:1:1, www) was solubilized in DDM too, and supplemented to the protein at a lipid-to-protein ratio (LPR) of 0.1, and then diluted with the protein buffer to a final protein concentration of 0.5 mg/ml. The protein-lipid-detergent mixture was dialyzed in cassettes (NMWL 10 kDa, ThermoFisher Scientific) at room temperature against 1 L of protein buffer without DDM (100 mM Tris-HCl at pH 7.6, 150 mM NaCl, and 20% glycerol) for 12 h. The proteo-liposomes were harvested from the cassettes after dialysis (sample). The reconstitutions were checked by negative-stain electron microscopy for the presence of protein-packed vesicles of intermediate size (200~500 nm, Supplementary Fig. 1). For experiments, the samples were diluted with the physisorption buffer containing 100 mM Tris-HCl at pH 7.6, 150 mM KCl, and 20 mM MgCl_2_, of which 2 ul was deposited onto freshly cleaved mica and incubated for 10 min for physisorption. The excess proteo-liposomes, not physisorbed to the mica, were rinsed with the imaging buffer containing100 mM Tris-HCl at pH 7.6 and 150 mM KCl. The physisorption was kept short, 10 min, to assure low sample density on the mica surface.

### Cryo-electron microscopy (Cryo-EM) and 2D-crystallographic analysis

3.5 µl of solution containing 2D crystals were applied to glow-discharged Quantifoil R1.2/1.3 grids for 1 min, blotted for 3 s and then vitrified by plunging into liquid nitrogen-cooled liquid ethane in a FEI Vitrobot Mark IV (FEI). Samples were transferred to an FEI Titan Krios and 2D crystals were imaged for 2 s in 100 ms frames at a dose of 1 electron per Å per second at 22,500x in super-resolution counting mode using a Gatan K3 direct electron detector. Images were corrected for drift using whole frame and patch algorithms and Fourier cropped using MotionCorr2^66^. Images were unbent and the best 8 images were merged using a lattice of *a* = *b* = 95 Å and γ = 90˚ using Focus^67^. The best 8 images based on merging phase residual were merged to calculate a projection map in layer group *p*42_1_2 with a 4 Å resolution limit.

### Lipid preparation

Lipids (1,2-dimyristoleoyl-sn-glycero-3-phosphocholine (C14), 1,2-dipalmitoleoyl-sn-glycero-3-phosphocholine (C16), 1,2-dioleoyl-sn-glycero-3-phosphocholine (DOPC, C18), and 1,2-dieicosenoyl-sn-glycero-3-phosphocholine (C20)) purchased from Avanti polar lipids (Supplementary Fig. 4) were solubilized in chloroform. The solubilized lipids were dried by a nitrogen flow and further dried in a vacuum chamber for 12 h. The dried lipids were resuspended in the imaging buffer (100 mM Tris-HCl at pH 7.6 and 150 mM KCl). The resuspended lipids were tip-sonicated for 2 min to obtain small unilamellar vesicles (SUVs). SUVs were used during the lipid addition step in the HS-AFM fluid cell for the membrane extension and fusion experiments (see main text: Experimental design to study membrane-mediated protein interactions).

### High-speed Atomic force microscopy (HS-AFM)

HS-AFM measurements were performed with a HS-AFM (RIBM) operated in amplitude modulation mode, using lab built amplitude detectors and force stabilizers^68^. Igor Pro version 6.37 was used for HS-AFM data collection. In brief, we used short cantilevers (USC-F1.2-k0.15, NanoWorld) with a nominal spring constant of 0.15 N m^–1^, resonance frequency of ~0.6 MHz and a quality factor of ~1.5 in the imaging buffer (100 mM Tris-HCl at pH 7.6 and 150 mM KCl). All data was acquired at room temperature.

### HS-AFM height spectroscopy (HS-AFM-HS)

HS-AFM-HS data was taken by disabling *x*- and *y*-scanning directly after HS-AFM imaging, as previous described^35^. In this mode, the tip is positioned at the center of the previous image with the z-feedback loop remaining active, monitoring the molecules diffusing in the membrane under the tip ~100 nm away from the closest AqpZ array. All measurements were taken with a free amplitude ~3 nm and a set-point amplitude of >90% of the free amplitude. Z-piezo data, 0 nm height was set to the membrane surface baseline (Fig. 2f, middle), was captured with home written software and a data acquisition board with a maximum acquisition rate of 2,000,000 samples s^−1^ (LabView programming, NI-USB-6366 card, National Instruments). All data was acquired at room temperature.

### Bilayer extension

SUVs of interest (C14, C16, C18 and C20, Supplementary Fig. 4) were diluted with the imaging buffer (100 mM Tris-HCl at pH 7.6 and 150 mM KCl) to a final lipid concentration of 1 mg/ml, of which 10 ul was added to the HS-AFM fluid chamber during HS-AFM imaging. Continuous HS-AFM imaging directly reported bilayer formation, extension and fusion with the membrane protein patches. Based on the low surface density, ~5% (Fig. 1f) and the low LPR = 0.1 of the reconstituted sample, we estimated that >99% of the lipids in each experiment are supplemented C14, C16, C18 or C20.

### Protein hydrophobic thickness determination

Protein hydrophobic thickness was determined with home written MATLAB scripts (MatLab, Mathworks). In brief, atom/residue coordinate data of the protein structure was obtained from the Protein Data Bank (PDB). First, the coordinates were normalized so that the center of the protein is at the origin of the coordinate system and the symmetry axis accords with the z-axis. Then the *x and y* coordinates were converted to their polar equivalents, *r* and *θ*, so that an atom can be characterized with three values: *r*, *θ* and *z*. Each pixel, *p*, of the 360° ‘unrolled’ structure surface plot can be characterized with two values in space: the polar angle θ, i.e. the position in a row, and *z*, i.e. the position in a column. All atoms within a region of defined size (height: 10 Å, angle: 10°) around each pixel were considered to score and determine its relative abundance in hydrophobic (red), hydrophilic (blue) and aromatic (green) surface exposed residues. The hydrophobic score, *R*_*p*_, is calculated as: ${R}_{p}=\sum ({\delta }_{{iR}}{{\exp }}(-\frac{{({z}_{i}-{z}_{p})}^{2}}{{\sigma_{z} }^{2}}){{\exp }}(\frac{{-({r}_{i}-{r}_{{{\max }}})}^{2}}{{\sigma_{r} }^{2}}){{\exp }}(\frac{{-({\theta }_{i}-{\theta }_{p})}^{2}}{{\sigma_{\theta} }^{2}}))$, where ${\delta }_{{iR}}$= 1 if atom *i* belongs to a hydrophobic residue and 0 otherwise, and *r*_*max*_ is the radius of the most exposed residue in the region. This equation gives higher scores to the surface exposed residues. Similarly, the hydrophilic score, *B*_*p*_, and aromatic score, *G*_*p*_, are calculated by substituting ${\delta }_{{iR}}$ with ${\delta }_{{iB}}$ and ${\delta }_{{iG}}$, respectively. The highest score among the three defines the pixel’s property, e.g. the pixel is a hydrophobic pixel and colored red when the hydrophobic score is highest. For the calculation of the hydrophobic thickness, we only consider the hydrophilicity and hydrophobicity. In particular cases, e.g. OmpF used here as test protein, aromatic residues form girdles around membrane proteins separating rather well defined hydrophobic and hydrophilic regions. The hydrophobic thickness *l* is determined as *l* = *A*_hydrophobic_*/c*_surface_, where *A*_hydrophobic_ represents the area of the hydrophobic pixels on the ‘unrolled’ surface and *c*_surface_ represents the surface width.

### HS-AFM data analysis

HS-AFM movies were aligned, flattened, and calibrated using home written ImageJ plugins (ImageJ, NIH). HS-AFM-HS data were analyzed with home written MATLAB scripts, as described^35^. For one-bond and two-bond events analysis, we picked and identified particles, i.e., cytoplasmic and extracellular proteins, in the protein array using a home written ImageJ plugin to obtain the coordinates of the array-bound proteins in each HS-AFM frame (time-resolved coordinates, see Supplementary Fig. 3). The time-resolved coordinates were then analyzed with home written MATLAB scripts for event sorting, dwell time counting, and fittings.

### Membrane protein automata

The membrane protein automata simulations were implemented as a custom written Python program, modified from the cellpylib package^69^. The simulations were analyzed using custom written MATLAB scripts, akin the experimental data analysis. See Supplementary Note 2 for the state-update rules and other details.

We write the membrane-dependent energy change of a membrane configuration rearrangement, *Δψ*, as (Eq. (7) in the main text):

$$\Delta \psi={\delta n}_{1}{\psi }_{1}+{\delta n}_{2}{\psi }_{2}+{\delta n}_{3}{\psi }_{3}+{\delta n}_{4}{\psi }_{4},$$

where *δn*_*i*_ is the change, gain or loss, of local-configuration *i* in the rearrangement. We solved through numerical simulations the 2D continuous elastic field *u*_*xy*_ of local-configurations 1 to 4 in Fig. 3f and determined *ψ*_*1*_ to *ψ*_*4*_. In the automata, we consider {*ψ*_*1*_ *ψ*_*2*_ *ψ*_*3*_ *ψ*_*4*_} = *ψ*_*1*_*ψ*_norm_, where *ψ*_norm_ = {*ψ*_*1*_ *ψ*_*2*_ *ψ*_*3*_ *ψ*_*4*_}/*ψ*_*1*_ relates the relative energies of the local configurations to each other. We first used a cylindrical protein model in the numerical simulation, which gave, on average, *ψ*_norm_ = {1.00 1.81 3.01 3.50} (Supplementary Fig. 7). Intuitively, gain of one local-configuration 2 costs two local-configuration 1 s, and in this scenario, configuration 2 with *ψ*_norm_ = 1.81 is favored, because *ψ*_*2*_*−2ψ*_*1*_ < 0. Accordingly, configuration 3 (3.01) is slightly unfavored, while configuration 4 (3.50) is strongly favored. Because the protein cross-section shape and orientation in the configurations matter for the 2D deformation field, we modeled AqpZ using a clover-leaf-like cross-section (Supplementary Fig. 7), based on the cryo-EM data (Supplementary Fig. 2). The numerical simulation gave, on average, *ψ*_norm_ = {1.00 2.06 3.22 4.10}. Thus, in both models, configuration 3 is unfavored relative to configurations 2 and 4, and therefore will be rare at equilibrium. For example, using Eq. (7), *Δψ* of the rearrangements in Fig. 3g are *Δψ*_re1_ = 0.4 and *Δψ*_re2_ = −0.5 using the cylindrical model, and *Δψ*_re1_ = 0.32 and *Δψ*_re2_ = 0.1 using the clover-leaf model. In both cases, the difference between the two rearrangements is *Δψ*_re2_-*Δψ*_re1_ < 0, explaining why arrays tend to have square shape.

For all simulations shown in Fig. 3h and supplementary Fig. 10, we used the shape-realistic *ψ*_norm_ = {1.00 2.06 3.22 4.10} and scaled the energies by *ψ/ψ*_norm_ to approximate the experimentally determined energy gain due to hydrophobic mismatch square (*u*_*0*_^2^). The membrane protein automaton generated protein arrays that displayed similar morphology as in the experiment (Fig. 3h). We also performed simulations using different *ψ*_norm_ favoring individual local configurations and found that the assembly morphology changed dramatically from fuzzy to square arrays (Supplementary Fig. 10d–f), or using different *ψ*/*ψ*_norm_ mimicking different hydrophobic membrane mismatch (Supplementary Fig. 10g–i).

### Reporting summary

Further information on research design is available in the Nature Portfolio Reporting Summary linked to this article.

## Supplementary information

Supplementary Information

Description of Additional Supplementary Files

Supplementary Movie 1

Supplementary Movie 2

Supplementary Movie 3

Supplementary Movie 4

Supplementary Movie 5

Supplementary Movie 6

Supplementary Movie 7

Supplementary Movie 8

Supplementary Movie 9

Supplementary Movie 10

Reporting Summary

## Source data

Source Data

## Data Availability

Data supporting the findings of this manuscript are available from the corresponding author upon request. The source data underlying all figures are available as a Source Data file provided with this paper. Source data are provided with this paper.

## Code availability

All codes used for data analysis may be requested from the authors.
