# Supplementary material for: Membrane-mediated protein interactions drive membrane protein organization
Source: Nat Commun. 2022 Nov 30;13:7373. doi: 10.1038/s41467-022-35202-8 (PMC9712761; doi:10.1038/s41467-022-35202-8)
Supplement: Supplementary file 1 — Supplementary Information [file 41467_2022_35202_MOESM1_ESM.pdf]

Membrane-mediated protein interactions drive membrane protein organization

Author list

Yining Jiang,[1,2] Batiste Thienpont,[3] Vinay Sapuru,[4] Richard K Hite,[4] Jeremy Dittman,[5] James N Sturgis,[3] Simon Scheuring[2,6,7,*]

Affiliations

[1] Biochemistry & Structural Biology, Cell & Developmental Biology, and Molecular Biology (BCMB) Program, Weill Cornell Graduate School of Biomedical Sciences, 1300 York Avenue, New York, NY-10065, USA.

[2] Weill Cornell Medicine, Department of Anesthesiology, 1300 York Avenue, New York, NY-10065, USA.

[3] Laboratoire d'Ingénierie des Systèmes Macromoléculaires (LISM), Unité Mixte de Recherche (UMR) 7255, Centre National de la Recherche Scientifique (CNRS), Aix Marseille Université, Marseille, France.

[4] Structural Biology Program, Memorial Sloan Kettering Cancer Center, 1275 York Avenue, New York, NY-10065, USA.

[5] Weill Cornell Medicine, Department of Biochemistry, 1300 York Avenue, New York, NY-10065, USA.

[6] Weill Cornell Medicine, Department of Physiology and Biophysics, 1300 York Avenue, New York, NY-10065, USA.

[7] Kavli Institute at Cornell for Nanoscale Science, Cornell University, Ithaca, New York 14853, USA.

* Correspondence to: sis2019@med.cornell.edu

Supplementary Information

Supplementary Figure 1

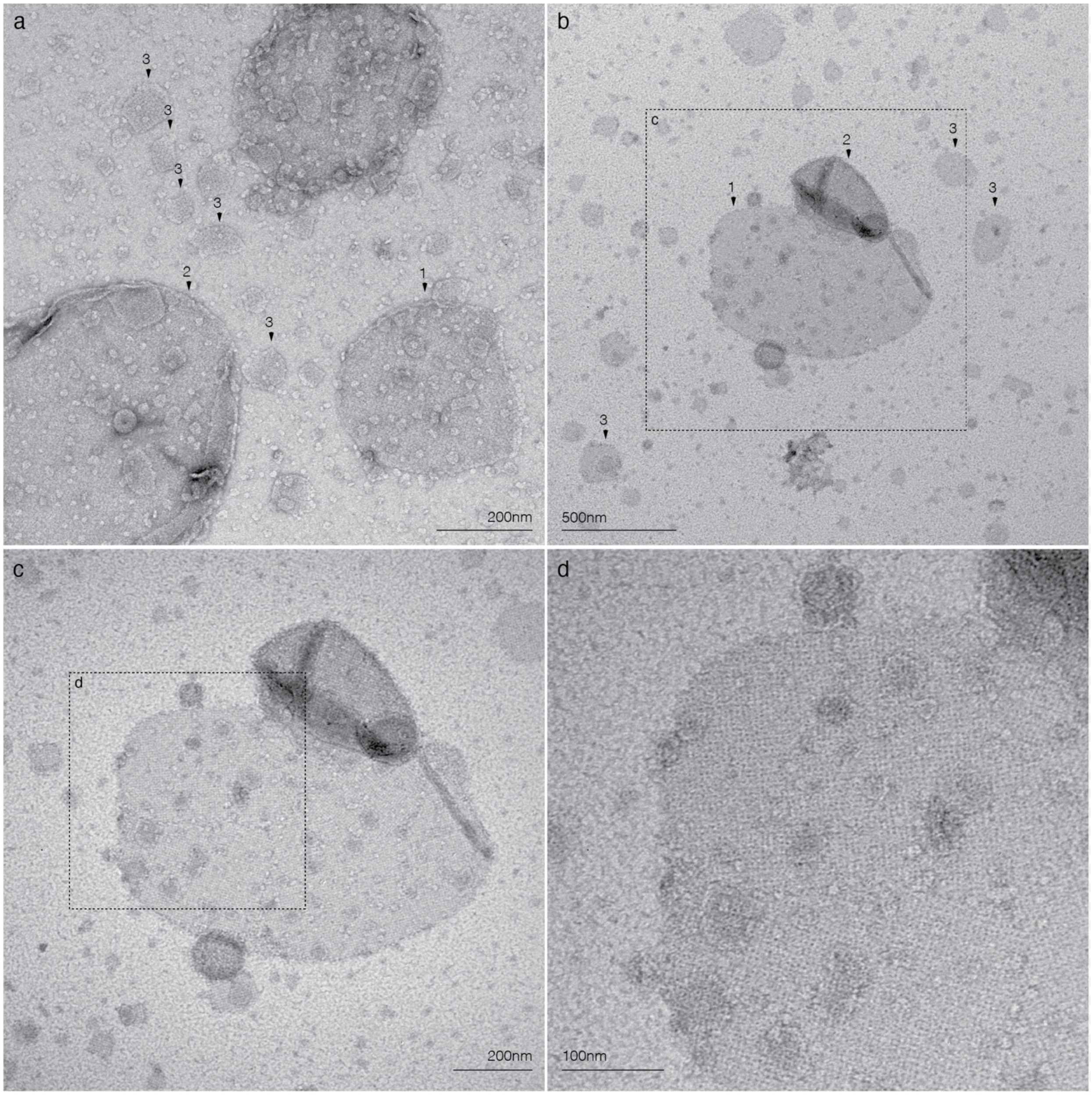

Supplementary Figure 1 | Negative stain electron microscopy (EM) of AqpZ-W14A reconstitution. (a) and **(b)** Micrographs with 2D-sheet (arrowhead 1), 2D-crystalline proteo-liposome (arrowhead 2) and several small 2D-sheets (arrowheads 3). **(c)** and **(d)** Zoom-in series of a 2D-sheet in (b). The 2D square lattice of AqpZ is discernible in (d). Similar results were obtained in all samples.

Supplementary Figure 2

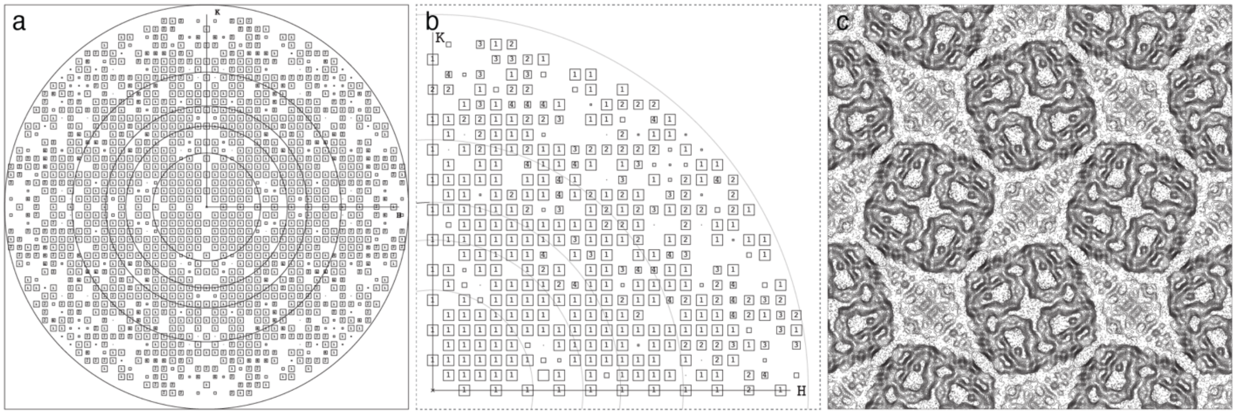

Supplementary Figure 2 | Cryo electron microscopy (cryo-EM) 2D-crystallographic analysis of AqpZ-W14A 2D-crystals. (a) IQ-plot of the merged 2D-projection data. The resolution rings (from the center to Nyquist of the plot) represent 15 Å, 10 Å, 8 Å, 6 Å and 4 Å resolution. The diffraction spots are marked with their respective figure of merit (FOM): 1, >95; 2, >90; 3, >85; 4, >80; 5, >75; 6, >70; 7, >65; 8, >60; 9, <60. The Fourier space crystal axes H and K are indicated. **(b)** Zoom-in image of the first (upper-right) qudrant in (a). **(c)** 4Å-projection structure of AqpZ-W14A. Four unit cells are shown (full image size 190 Å). The plane-group symmetry of the 2D-crystal is $p42_12$.

Supplementary Figure 3

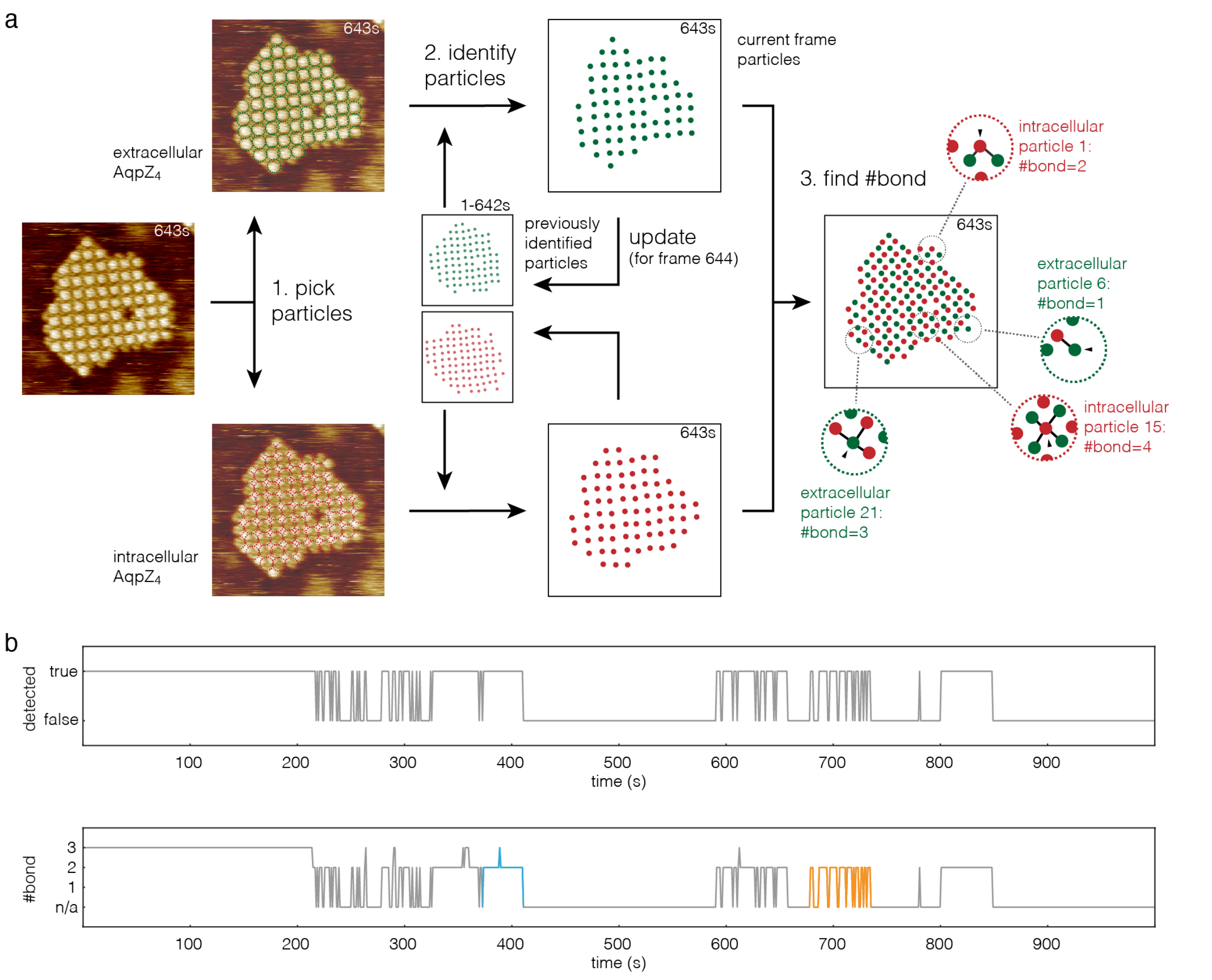

Supplementary Figure 3 | Workflow of the AqpZ association/dissociation events analysis. (a) The workflow of the AqpZ association/dissociation events analysis. Single particles, extracellular (green) and intracellular (red), were picked from each HS-AFM frame (time) to extract the coordinates (*step 1*). The picked particles were given an identifier by coordinates comparison with the previously identified particles (*step 2*), in which process newly identified particles were updated for later frames (*update*). Both extracellular (E) and intracellular (C) particles were combined for the assignment of the number of bonds (#bond) for each particle (*step 3*), where each particle is assigned the idealized lattice position closest to the location in which it is detected. As diffusing molecules are not detected in HS-AFM imaging, all resolved particles are assumed part of the lattice. Four exemplary single particles (arrowheads) and their bonds (dark lines) are displayed. **(b)** Time-evolution changes of the detection (top) and #bond (bottom) for an example single particle. If the particle is not detected in a frame, *N/A* is assigned to #bond of that frame. From these plots, dwell times of complete events (association and dissociation with unchanged environment) are extracted. Examples: Blue: Not a complete event, due to change of #bond, *i.e.* change of molecular environment. Orange: Series of two-bond events. A false detection in the top time sequence corresponds to n/a in the bottom sequence. **(c)** Time-evolution changes of #array-bound molecules.

Supplementary Figure 4

14:1 (Δ9-Cis) PC
1,2-dimyristoleoyl-sn-glycero-3-phosphocholine
Synonym: 1,2-di-(9Z-tetradecenoyl)-sn-glycero-3-phosphocholine; PC(14:1(9Z)/14:1(9Z))
Molecular Weight: 673.90
Phase Transition Temperature: -51.4°C*

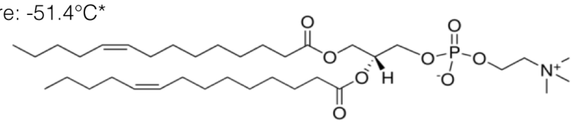

16:1 (Δ9-Cis) PC
1,2-dipalmitoleoyl-sn-glycero-3-phosphocholine
Synonym: 1,2-di-(9Z-hexadecenoyl)-sn-glycero-3-phosphocholine; PC(16:1(9Z)/16:1(9Z))
Molecular Weight: 730.007
Phase Transition Temperature: -36°C

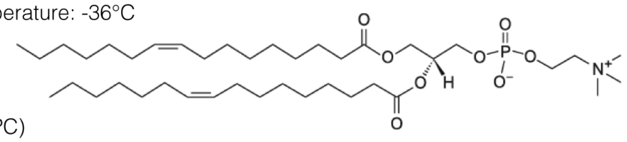

18:1 (Δ9-Cis) PC (DOPC)
1,2-dioleoyl-sn-glycero-3-phosphocholine
Synonym: 1,2-di-(9Z-octadecenoyl)-sn-glycero-3-phosphocholine; PC(18:1(9Z)/18:1(9Z))
Molecular Weight: 786.113
Phase Transition Temperature: -17°C

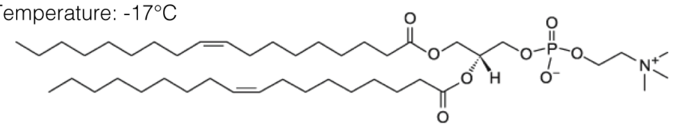

20:1 (Cis) PC
1,2-dieicosenoyl-sn-glycero-3-phosphocholine
Synonym: 1,2-di-(11Z-eicosenoyl)-sn-glycero-3-phosphocholine; PC(20:1(11Z)/20:1(11Z))
Molecular Weight: 842.220
Phase Transition Temperature: -4.3°C

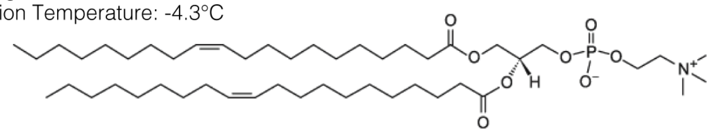

Supplementary Figure 4 | Chemical structures and properties of the lipids used in the experiments. All lipids have the same head group and the same degree of saturation, namely one cis-double bond roughly at the mid-position of the hydrocarbon tail, *i.e.* Δ9 position for C14, C16 and C18 lipids and at Δ11 position for C20 lipids. These lipids all have melting temperatures <0 °C, thus are in liquid phase throughout the experiments (room temperature). These structural similarity of the lipids, where the most significant difference resides in the length of the hydrocarbon tails, ensures that the observed difference in the membrane-mediated membrane protein interactions can be related to the hydrophobic thickness of the bilayers.

Supplementary Figure 5

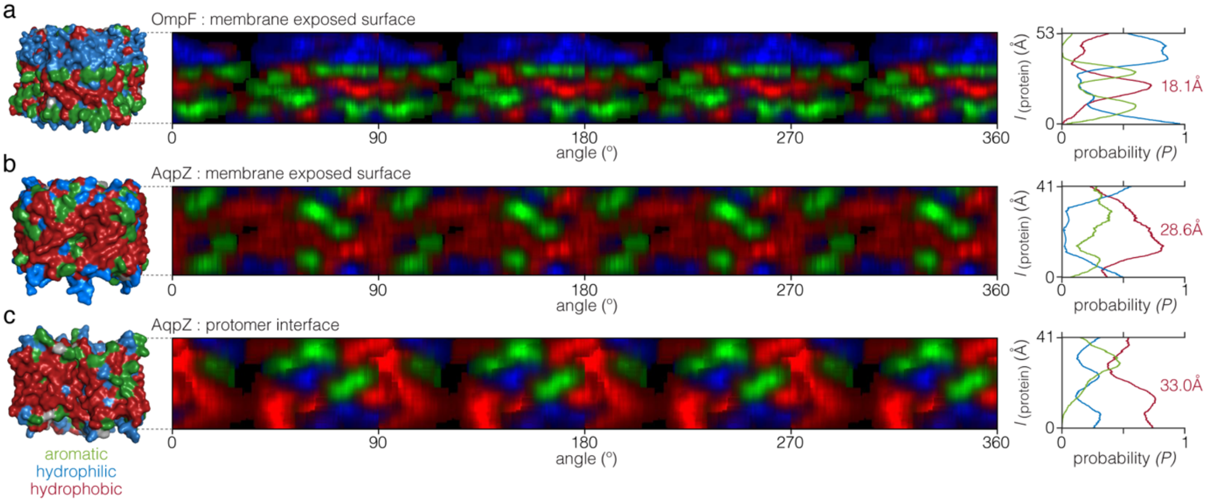

Supplementary Figure 5 | Analysis of membrane protein hydrophobic thickness. In all panels: Blue: hydrophilic residue surface, red: hydrophobic residue surface, green: aromatic residue surface (indicated below the structures). (**a**) OmpF, membrane exposed surface (PDB 2OMF), (**b**) AqpZ, membrane exposed surface (PDB 2O9D), and (**c**) AqpZ, protomer interface (PDB 2O9D). From left to right: Surface representation of the structure, 360º 'unrolled' surface of the structure, and plot of the relative abundance of hydrophilic, hydrophobic and aromatic surface exposed residue surfaces along the protein thickness. The hydrophobic thickness l is determined as $l = A_{hydrophobic}/c_{\mathrm{surface}}$, where $A_{hydrophobic}$ represents the area of the hydrophobic pixels on the 'unrolled' surface and c_{surface} represents the width of the 'unrolled' surface.

Supplementary Figure 6

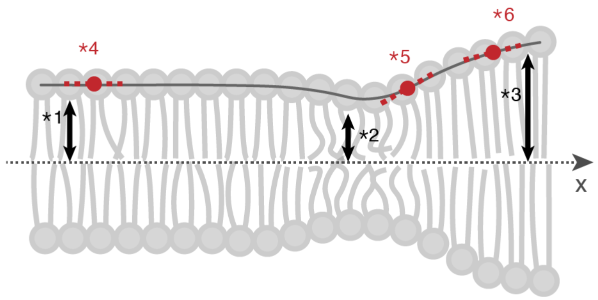

Supplementary Figure 6 | Schematic illustration of membrane compression and bending. Membrane compression (black arrows) is defined as the change of local lipid leaflet thickness compared to the resting leaflet thickness, thus the local hydrophobic mismatch. No compression (asterisk 1), positive compression (asterisk 2) and negative compression (asterisk 3) are shown. The red dashed lines indicate the tangents of the local slopes, *i.e.* 1st derivative of the hydrophobic mismatch. Membrane bending is defined as the change in local slope (red dots), thus the 2nd derivative of local hydrophobic mismatch. No bending (asterisk 4, tagent holds), positive bending (asterisk 5, tangent increases) and negative bending (asterisk 6, tangent decreases) are displayed. The schematic illustrates a single type of lipid. Schematic generated using Biorender.com..

Supplementary Figure 7

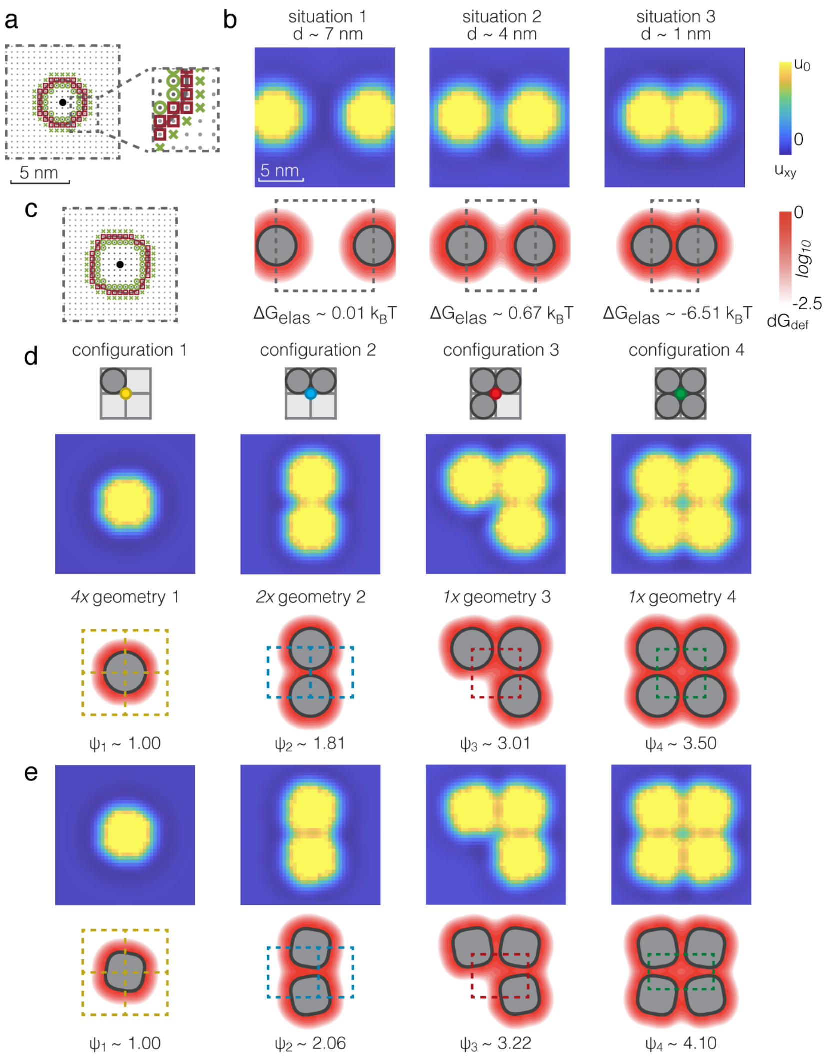

Supplementary Figure 7 | 2D membrane deformation fields and energies. (**a**) and (**b**) The 2D membrane deformation of two cylindrical membrane proteins. (**a**) The model built for a cylindrical membrane protein on a grid of nodes. Each node represents a 0.5 nm x 0.5 nm area on a discretized membrane deformation field. Each cylindrical membrane protein has a cross-section radius of 2 nm. Black dot: Center of mass (COM). Red squares: Boundary nodes. Green circles: Interior boundary nodes. Green crosses: Exterior boundary nodes. (**b**) Three numerically simulated situations of two cylindrical membranes at 7 nm (Left), 4 nm (Middle), and 1 nm (Right). Distance *d* is defined as the distance between protein COMs minus two times the protein radius (edge-to-edge distance). The membrane deformation fields, u_{xy}, is solved (Top) using finite difference method (see Supplementary Note 1), from which the deformation energy density map, dG_{def}, is determined (Bottom). In the simulation, $u_0 = 0.2$ nm and $l = 1.2$ nm (C14 lipid). The deformation field within the protein boundary is meaningless and filled with u_0 for illustration purpose. The elastic potential between the proteins, ΔG_{elas}, is calculated by the integration over the selected area on the energy density map (dash lines) minus the area occupied the protein. (**c**) The clover-leaf AqpZ model based on the Cryo-EM data (Supplementary Fig. 2, Supplementary Note 1). $R = 2.6$ nm, $\varepsilon = 0.006$ and $\omega = \pm 30°$. (**d**) and (**e**) The 2D deformation fields (Top) and the deformation energy density maps (Bottom) of the four local-configurations using: (**d**) Cylindral protein model as shown in (a). $R = 2.62$ nm. (**e**) Clover-leaf AqpZ model as shown in (c). In both (d) and (e), the distance between COMs is 9.5 nm, and the closest edge-to-edge distance is ~1 nm. The integration area (dash lines) is confined with boundary lines either crossing where the two neighbor molecules are closest, ~1 nm, or the protein COMs. Integrations over the selected areas give, from left to right: 4x ψ_1, 2x ψ_2, 1x ψ_3, and 1x ψ_4.

Supplementary Figure 8

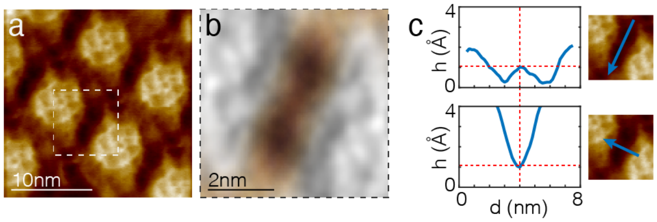

Supplementary Figure 8 | Saddle-shaped membrane deformation between proteins. (a) AqpZ array (time average over 34 frames). White box: membrane area encircled by four AqpZ tetramers. **(b)** LAFM map of the highlighted region in (a). **(c)** Line profiles of the highlighted region in (a). h: z-values . d: Distance along the arrows in the insets (right). The characterization of the saddle-shaped membrane area between proteins must be considered with caution as only very sharp tips can probe the narrow region between proteins.

Supplementary Figure 9

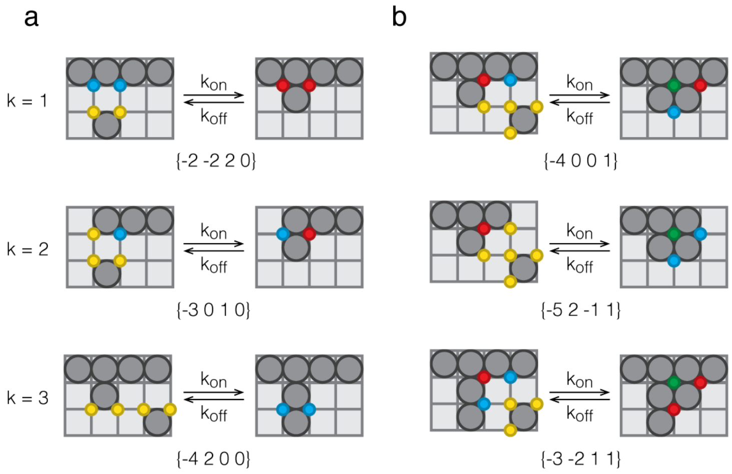

Supplementary Figure 9 | Rearrangements of the membrane configurations in association/dissociation events. With association/dissociation events to and from states *1B* **(a)** and *2B* **(b)**, the 2D membrane local-configuration changes and thus energetic changes occur. The changes of the local-configurations are displayed as $\{\delta n_1\ \delta n_2\ \delta n_3\ \delta n_4\}$ below the rearrangements.

Supplementary Figure 10

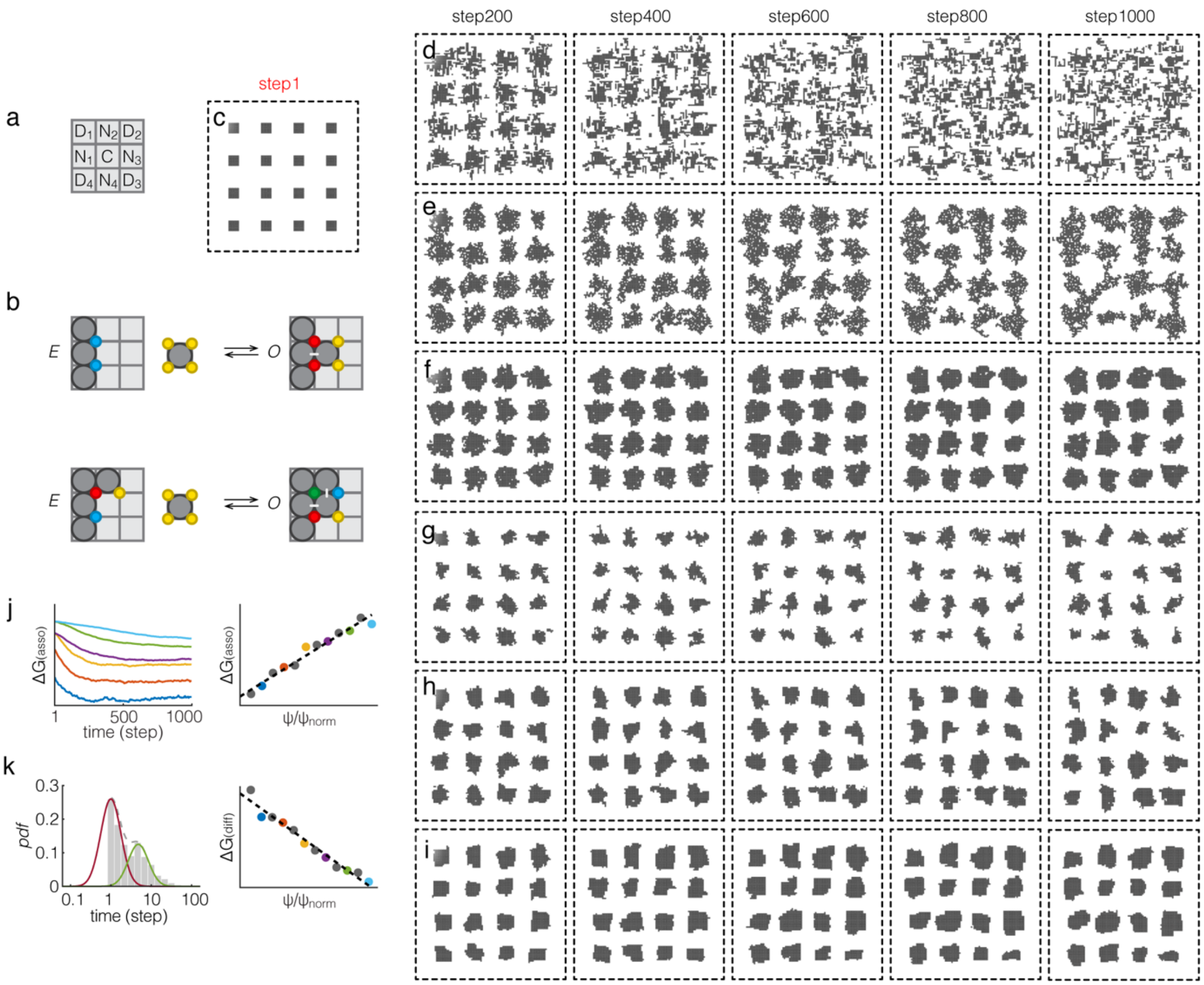

Supplementary Figure 10 | Membrane protein automata. (**a**) The environment of a membrane protein in the membrane protein automaton. The environment of a membrane protein is represented as a 3x3 kernel, including itself in the central field (*C*), its four direct neighbors (N_1-N_4), and the four diagonal neighbors (D_1-D_4). (**b**) Each field in the automaton is either occupied (state *O*) or empty (state *E*). Examples of a one-bond event (top) and a two-bond event (bottom) are given for state update rules illustration (see Supplementary Note 2). Local-configurations (see figure 3) and bonds (white sticks) are marked. (**c**) The initial state of the automata: 1024 (16*8x8) out of 13225 (115x115) fields are at state *O*. Different intial concentration of diffusing molecules (C_{init})was used in each simulation to ensure the system reaching equilibrium (see Methods and Supplementary Note 2). (**d**), (**e**) and (**f**) Membrane protein automata with imaginary ψ_{norm} (see text) favoring local-configuration *2* (d, ψ_{norm} = {1.00 1.90 3.00 4.00}), local-configuration *3* (e, ψ_{norm} = {1.00 2.00 2.90 4.00}), and local-configuration *4* (f, ψ_{norm} = {1.00 2.00 3.00 3.90}). (**g**) to (**l**) Membrane protein automata with ψ_{norm} = {1.00 2.06 3.22 4.10} and analysis (see text). (**g**), (**h**) and (**i**) Selected frames of membrane protein automata in membranes of no (g), small (h), and large (i) hydrophobic mismatch. (**j**) The time-evolved macroscopic association energy ΔG_{macro} (left, see definition in Supplementary Note 2) and ΔG_{macro} at equilibrium (steps 901-1000) as a function of the energy scale factor ψ/ψ_{norm} (right, also shown as Fig. 3i). (**k**) The energy difference between states *1B* and *2B* (ΔG_{diff}). Left: The probability density function (*pdf*) of the dwell times (n = 19417) in one automaton, displayed in log-binning and fitted with two gaussians, representing the time constants. Right (also shown as Fig. 3j): ΔG_{diff} as a function of the energy scale factor (ψ/ψ_{norm}).

Supplementary Figure 11

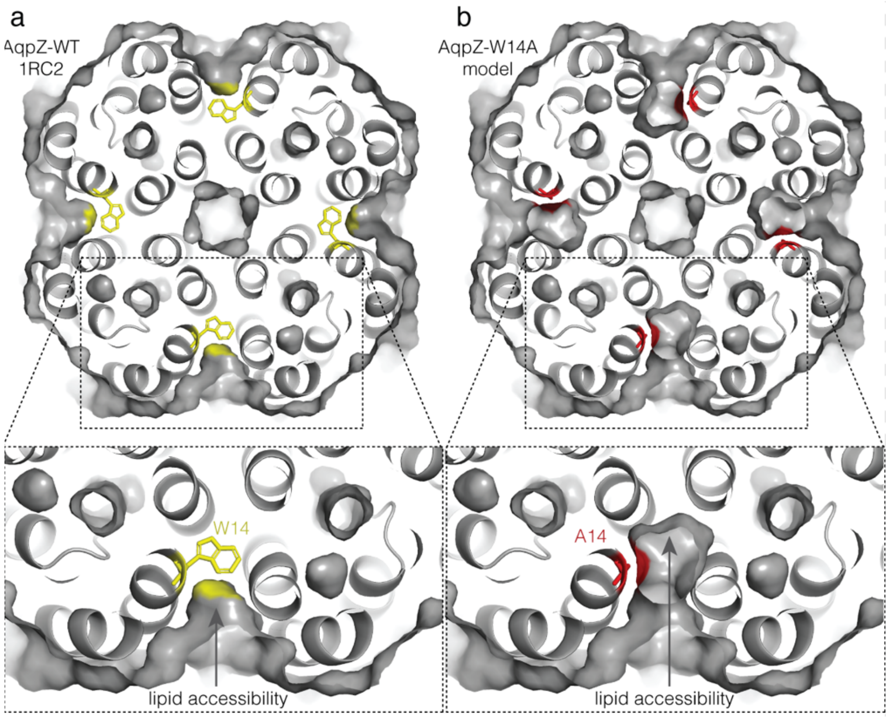

Supplementary Figure 11 | The AqpZ-W14A mutation. Slices approximately mid-membrane through the AqpZ tetramer (**a**) WT and (**b**) W14A (model). The substitution of the bulky W14 in the AqpZ-WT (yellow) to the small A14 in AqpZ-W14A appears to open a cavity for lipids to intercalate between the protomers (arrows).

Supplementary Figure 12

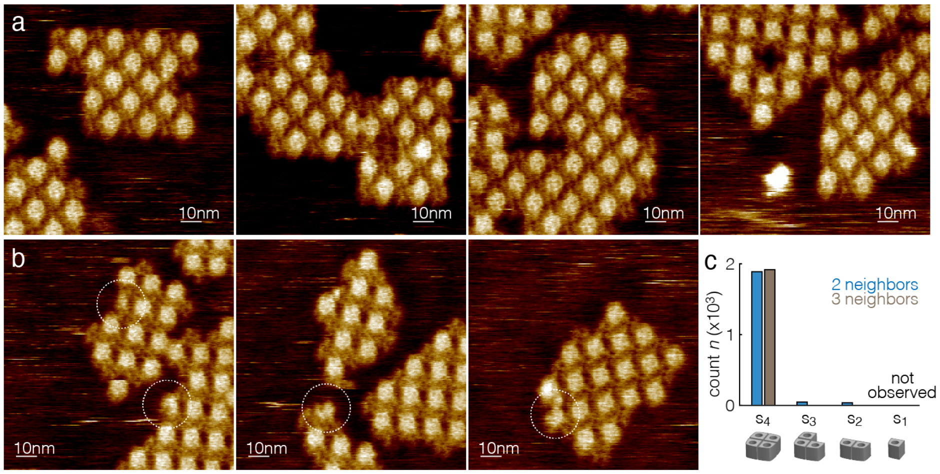

Supplementary Figure 12 | The AqpZ WT protomer association and dissociation dynamics in a lipid bilayer that matches the hydrophobic thickness of the AqpZ protomer-protomer interface. (a) and **(b).** HS-AFM movie frames of AqpZ WT oligomers in a C20 membrane (image parameter: 0.33 nm/pixel): (a) Regions where all oligomers were intact, $AqpZ_4$. (b) Regions where non-canonical AqpZ oligomers, $AqpZ_2$ and $AqpZ_3$, were observed (dashsed circles). **(c)** Occurrence probabilities of AqpZ WT oligomeric states at the array edge.

Supplementary Note 1: 2D deformation fields and energies

The continuous elastic field, u_{xy}, is the deviation of each lipid head-group from its unperturbed height as a function of space, xy, in a 2D cartesian coordinate. The expression of G_{def} is[1]:

$$G_{def} = \frac{1}{2}\int\int\left[K_A\left(\frac{u_{xy}}{l}+\frac{\tau_E}{K_A}\right)^2 + \kappa_b\left(\nabla^2 u_{xy} - c_0\right)^2\right]dxdy\ , \qquad \text{(S2.1)}$$

where $\nabla^2 = \frac{\partial^2}{\partial x^2}+\frac{\partial^2}{\partial y^2}$ is the Laplacian operator, and K_A is the bilayer stretch modulus, l the thickness, κ_b the bending modulus, τ_E the external tension, and c_0 the spontaneous curvature. Minimization of G_{def} under the boundary conditions, dependent on the geometries of the membrane and protein configuration, gives u_{xy} that characterizes the membrane deformation. The boundary conditions specify that the hydrophobic regions of the bilayer core and the protein TMD outer surface must be matched at the protein-lipid interfaces, and the slope at the protein-lipid interfaces is zero[1]. In this paper, the hydrophobic region of the protein TMD outer surface is considered having constant height at all directions and free of fluctuation, thus at the protein-lipid interfaces, $u = u_0$ and $|\nabla u| = 0$. In the HS-AFM experiment, the membrane is considered free of external tension and spontaneous curvature, thus *eq. S2.1* becomes *eq. 6* in the main text:

$$G_{def} = \frac{1}{2}\int\int\left[K_A\left(\frac{u_{xy}}{l}\right)^2 + \kappa_b\left(\nabla^2 u_{xy}\right)^2\right]dxdy\ . \qquad (5)$$

Eq.5 was numerically minimized to solve for u_{xy}[2]. In short, minimiazation of *eq.5* is equivalent to solving the Euler-Lagrange equation:

$$\kappa_b \nabla^4 u_{xy} + \frac{K_A}{l^2}u_{xy} = 0\ . \qquad \text{(S2.2)}$$

We used the finite difference (FD) method to solve *eq. S2.2* by discretizing the continuous field into a grid of nodes. Node size h = 0.5 nm, corresponding to the size of one lipid molecule, was used in the numerical simulation (**Supplementary Fig. 7a**). We used the clover-leaf model as a simple coarse-grained approximation to the cross-sections of membrane proteins:

$$C(\theta) = R\{1+\epsilon\cos(\theta-\omega)\}\ , \qquad \text{(S2.3)}$$

where $C(\theta)$ is the cross-section in radial coordinate with the origin positioned at the center of mass (COM) of the protein, and R is the protein radius, ε the magnitude of the deviation of the protein cross section from the circle (ε = 0 for a cylindral protein) , and ω the tilt angle of the protein as compared to vertical axis (θ = 0). Nodes corresponding to the protein boundary, as well as the interior and exterior boundaries were identified.

Such, the deformation field, u_{xy}, is charactereized as a vector, **u**, and *eq. 5* can be written in its matrix format as[2]:

$$G_{def,FD} = \boldsymbol{u}^T\boldsymbol{Q}\boldsymbol{u}\ , \qquad \text{(S2.4)}$$

and

$$\boldsymbol{Q} = h^2\left(\frac{\kappa_b}{2}\boldsymbol{L}^T\boldsymbol{L} + \frac{K_A}{2l^2}\boldsymbol{I}\right)\ , \qquad \text{(S2.5)}$$

where $\boldsymbol{L}$ and $\boldsymbol{I}$ are Laplacian and identity matrices respectively. And *eq. S2.2* becomes:

$$\boldsymbol{Q}\boldsymbol{u} = \boldsymbol{v}\ , \qquad \text{(S2.6)}$$

where vector $\boldsymbol{v}$ contains zeros except for the rows corresponding to the protein boundary nodes. Besides, we also adjusted the rows corresponding to the protein boundary nodes in matrix $\boldsymbol{Q}$ to ensure the protein boundary nodes in $\boldsymbol{u}$ all have values of u_0. *Eq S2.3* was solved in MATLAB using the Jacobi iteration method[3]. In each iteration, we adjusted the rows in $\boldsymbol{u}$ corresponding to the interior and exterior nodes so that each exterior node has the same value as its closest interior counterpart. This roughly ensures the zero-slope requirement at the protein-lipid interfaces.

Using the workflow described above, we first solved the deformation fields and eneriges in which two identifical cylindral proteins (R = 2 nm) are positioned at different edge-to-edge distances d (**Supplementary Fig 7b**). The cylindral protein was set to have the same hydrophobic TMD core thickness as an AqpZ, and membranes of different thicknesses, corresponding to C14, C16, C18 and C20, were simulated. We used $K_A = 60\ k_B\text{T nm}^{-2}$ and a well-established relationship $\kappa_b = K_A l^2/12$ to characterize the membrane physical properties in all simulations[4,5]. The changes of the deformation energies, *i.e.* the elastic potential (ΔG_{elas}, *eq. 6*), when the proteins come closer to each other were compared (**Fig. 3b-e**). Aside of the discussion in the main text, we also observed that in a thicker membrane, *e.g.* C20, the elastic potential becomes attractive at longer distance, $d \sim 3.8$ nm, than in a thiner membrane, *e.g.* C14, where the potential becomes attractive at $d \sim 3$ nm. This is primarily due to the increased contribution from the membrane bending component, *i.e.* κ_b scales up with l, and the decreased contribution from the membrane compression component, *i.e.* K_A scales down with l (*Eq. 5*).

Then, we solved u_{xy} of local-configurations 1-4 (**Fig.3f**) using both the cylindral protein model ($\varepsilon = 0$, R = 2.62 nm, **Supplementary Fig. 7a**) and the clover-leaf AqpZ model ($\varepsilon = 0.06$, R = 2.6 nm, $\omega = \pm 30°$, **Supplementary Fig. 7c**). In local-configurations 2-4 built with both models, the COM between neighboring proteins is 9.5 nm and the closest edge-to-edge distance is 1 nm. We denote ψ_i as the minimized G_{def} under the boundary conditions given by the geometries of the membrane and protein configuration (**Supplementary Fig. 7d,e**). We consider $\{\psi_1\ \psi_2\ \psi_3\ \psi_4\} = \psi_1\psi_{norm}$, where $\psi_{norm} = \{\psi_1\ \psi_2\ \psi_3\ \psi_4\}/\psi_1$ shows the relative energies of the local-configurations. The numerical simulations using the cylindral protein model gives $\psi_{1(C14)} \sim 7.9$, $\psi_{1(C16)} \sim 0.85$, $\psi_{1(C18)} \sim 0.58$, $\psi_{1(C20)} \sim 5.2$ and $\psi_{norm} = \{1.00\ 1.81 \pm 0.05\ 3.01 \pm 0.13\ 3.50 \pm 0.26\}$ (*mean* ± *std*). The numerical simulations using the cylindral protein model gives $\psi_{1(C14)} \sim 7.3$, $\psi_{1(C16)} \sim 0.78$, $\psi_{1(C18)} \sim 0.54$, $\psi_{1(C20)} \sim 4.9$ and $\psi_{norm}=\{1.00\ 2.06 \pm 0.08\ 3.21 \pm 0.17\ 4.09 \pm 0.32\}$. Both simulations suggest that local-configuration 3 is unfavored as compared to local configuration 2 and 4, meaning that it is energetically favorable to eliminate this configuration (see main text). Besides, ψ_2-ψ_4 are larger when the proteins were built with the clover-leaf model, as compared with the cylindral model. This suggests that the geometry of the AqpZ may play a negative role in the stability of the AqpZ arrays. We deduce that much less dissociation events would have been observed if the AqpZ geometry was closer to the cylindrical model. Thus, the protein geometry is essential in the membrane-mediated array-formation process.

We noticed larger variance of the deformation energy in the more complex local-configuration involving more proteins, *i.e.* $std(\psi_4) > std(\psi_3) > std(\psi_2)$. Especially, $\psi_4/\psi_1 = 4.09 \pm 0.32$ in the clover-leaf model simulation, which means that this configuration is strongly favored, $\psi_4 << 4\psi_1$, in some membranes and strongly unfavored in the others, $\psi_4 >> 4\psi_1$. Since no significant difference of the array morphology was experimentaly observed in the investigated membranes, the real variance in ψ_2-ψ_4 is thought to be smaller. One explanation is that as the configuration becomes more complex and involves more proteins, the fluctuations in the hydrophobic region of protein TMD outer surface, *e.g.* AqpZ (**Supplementary Fig. 5b**), which also induce local membrane curvature and tension, may not be neglectble in solving and comparing G_{def} of these complex configurations. We think the averaged ψ_{norm} reflects the relative energies of the local-configurations, and the difference in the averaged ψ_{norm} as the protein geometry was modeled differently shows the trend in which the clover-leaf shape of AqpZ is inclined to destabilize the array. Thus, the averaged $\psi_{norm} = \{1.00\ 2.06\ 3.21\ 4.09\}$ was used in the membrane protein automata to simulate the array morphology and the dyanmics of the association/dissociation events at the array edges (**Supplementary Note 2**).

Supplementary Note 2: Membrane protein automata

We developed the membrane protein automata (based on the well-established cellular automata[6]) to simulate the array dynamics and morphology to complement the discretized framework to understand the membrane-mediated membrane protein interactions (**Supplementary Fig. 10**). A membrane protein automaton is composed of a grid of fields, equivalent to cells in cellular automata, each in one of two states: state *O* for occupied and state *E* for empty. Fields at state *E* are considered the diffusion field where probability of meeting an unbound molecule P_U (unit %) characterizes the concentration of freely diffusing molecules. Besides, all interactions are local, only dependent on the environment defined by a 3x3 kernel surrounding the field of interest, including the field of interest in the center (*C*), its four direct neighbors (N_1 to N_4), and four diagonal neighbors (D_1 to D_4) (**Supplementary Fig. 10a**). Direct protein-protein interaction, represented as *C-N*, with strength $E_{P\text{-}P}$, can be formed between *C* and one of its direct neighbors. Membrane-mediated membrane protein interactions are the result of the rearrangements of the membrane local-configurations 1-4, with strength ψ_1 to ψ_4 , respectively. These interactions are represented as *CNND*, *e.g.* $CN_1N_2D_1$ characterizes the upper-left membrane of the environment, the intersection point of fields *C*, N_1, N_2, and D_1 (**Supplementary Fig. 10a**). Thus, a 3x3 environment kernel includes four potential *C-N* sites and four *CNND* sites. In the example shown, $CN_1N_2D_1$ and $CN_1N_4D_4$ have local-configuration 2 at state *E* and local-configuration 3 at state *O*; The intersection points $CN_2N_3D_2$ (upper right) and $CN_3N_4D_3$ (lower right) have local-configuration 0, *i.e.* empty membrane, at state *E* and local-configuration 1 at state *O*; and there is a $C\text{-}N_1$ interaction at state *O* (**Supplementary Fig. 10b**, top).

We can write the energy of a state (*st* = *O* or *E*) through:

$$E_{total}^{st} = \psi^{st} + E_{P-P}^{st} \quad , \tag{S3.1}$$

$$\psi^{st} = n_1^{st}\psi_1 + n_2^{st}\psi_2 + n_3^{st}\psi_3 + n_4^{st}\psi_4 \quad , \tag{S3.2}$$

$$E_{P-P}^{st} = n_{P-P}^{st} E_{P-P} \quad , \tag{S3.3}$$

where *eq. S3.1* defines the total energy of a state, *st*, as the sum of the membrane local-configuration rearrangements described by *eq. S3.2* and the direct protein-protein interactions described by *eq. S3.3*, with n_i^{st} being the number of local-configuration *i* and n_{P-P}^{st} is the number of direct protein-protein interactions in the environment kernel. The multiplicity of state *st* is:

$$\Omega_{st} \propto e^{-E_{total}^{st}/k_B T} \quad , \tag{S3.4}$$

In the examples given, $\{n_1^E\ n_2^E\ n_3^E\ n_4^E\ n_{P-P}^E;\ n_1^O\ n_2^O\ n_3^O\ n_4^O\ n_{P-P}^O\}$ equals {4 2 0 0 0; 2 0 2 0 1} (**Supplementary Fig. 10b**, top) and {5 1 1 0 0; 1 1 1 1 2} (bottom).

Each field in the automaton is given an initial state by the user. After initialization, the automaton scans through the 3x3 kernel environment of each field of interest *C* in the current step *s*, and updates the state for the next step *s+1* following the state-update rules:

1. If all direct neighbors *N*s of *C* are occupied in the current step, the state does not change in the next step, which gives: $N_1 = N_2 = N_3 = N_4 = O \rightarrow C_{s+1} = C_s$.
2. Else if all direct neighbors *N*s of *C* are empty in the current step, *C* must be empty in the next step, *i.e.* the membrane protein, if there is any, must diffuse away. This gives: $N_1 = N_2 = N_3 = N_4 = E \rightarrow C_{s+1} = E$.
3. Else, the probabilities of the state of *C* in the next step given the current state of *C* ($P_{C_s \rightarrow C_{s+1}}$), are considered for state-updating, through analyzing the potential environments of *C*. W

$$P_{C_s \rightarrow C_{s+1}} = \left(\frac{\Omega_{C_{s+1}}}{\Omega_{O_{s+1}} + \Omega_{E_{s+1}}}\right)\left[1 - \delta_{C_s E}\,\delta_{C_{s+1} O}(1 - P_U)\right] \quad , \tag{S3.5}$$

The δ functions in *eq. S3.5* assures that if C is empty in the current step and occupied in the next step, *i.e.* $C_s = E$ and $C_{s+1} = O$, the probability of meeting an unbound molecule in the diffusion field, P_U, is considered. Besides, an additional diffusing molecule, having $\{n_1\ n_2\ n_3\ n_4\ n_{P\text{-}P}\} = \{4\ 0\ 0\ 0\ 0\}$, must always be included at state E to correctly account for the energy difference between states E and O (**Supplementary Fig. 10b** state E). This setup allows us to simulate distinct array dynamics and morphology through different combinations of $E_{P\text{-}P}$, P_U and $\psi = \{\psi_1\ \psi_2\ \psi_3\ \psi_4\}$. Notably, the ratios between the four ψ_i values define the likelihood of forming certain local-configuration, and the magnitudes, competing with the direct protein-protein interactions, define the strength of the membrane-mediated interactions.

In HS-AFM imaging, we observed that the association energy is lower in lipids with a small hydrophobic mismatch, while the energy between states *1B* and *2B* is lower in lipids with a large hydrophobic mismatch, both scaling linearly to the mismatch square. Thus, to imitate the hydrophobic mismatch, we fixed the ratios between the ψ_i values as ψ_{norm} (set $\psi_1 = 1$) and increased/decreased the magnitudes of the deformation energy through the scale factors ψ/ψ_{norm}, which can be interpreted as increasing/decreasing the hydrophobic mismatch square in the automata. We initialized the system with 16 8x8 (1024) square-shaped arrays in a 115x115 (13225) grid, thus 1024 of 13225 fields are at state O (**Supplementary Fig. 10c**) and simulated for 1000 steps. The total number of molecules, array-bound and diffusing, is fixed in each simulation as $N_{total} = 1024 + 13225 * P_U$, the former representing array-bound molecules and the latter diffusing molecules. The value of P_U is different in each simulation to ensure that the arrays grow within the grid before reaching equilibrium. For illustration, we also performed simulations with ψ_{norm} values favoring individual local-configurations (**Supplementary Fig. 10d,e,f** and **Supplementary Movie 8**, bottom row). These simulations suggested that the array morphology depends on the choice of ψ_{norm} values: Linear arrays dominate if configuration 2 is favored; Round-shaped and hollow arrays dominate if configuration 3 is favored; Square-shaped arrays dominate if configuration 4 is favored. We used $\psi_{norm} = \{1.00\ 2.06\ 3.22\ 4.100\}$ (**Supplementary Fig. 7** and **Supplementary Note 1**) in the automata to simulate AqpZ array dynamics (**Supplementary Fig. 10g,h,i** and **Supplementary Movie 8**, top row). Most of the arrays in these automata had a round-shaped morphology, which agrees with the observation made in the experiment (**Fig. 2a,b,c**). This preferred morphology can be explained by the unfavored local-configuration 3 in ψ_{norm}, comparing to other local-configurations (see text).

We then analyzed the arrays in the automata as described in the main text, and calculated ΔG_{asso} using *eq. 3*. The time-evolved ΔG_{asso} in all automata reached equilibrium after step 900 (**Supplementary Fig. 10j**, left). The ΔG_{asso} at equilibrium (average ΔG_{asso} over steps 901-1000) were compared in automata of different ψ/ψ_{norm} (right, also shown as **Fig. 3j**), suggesting that the hydrophobic mismatch undermines the association of a diffusing molecule to the array edges. Following, we collected 19417 complete events from one automaton and plotted the dwell times with the log binning method (**Supplementary Fig. 10k,** left). Two peaks were observed from this dwell time analysis, which supports the choice of two effective time constants in the kinetic model as a satisfactory approximation. Finally, we analyzed the dwell-times in the simulations, as previously described, and calculated ΔG_{diff} using *eq. 4*. The same membrane-dependent trend in ΔG_{diff} observations was also reproduced in the membrane protein automata (right, also shown as **Fig. 3k**), in which hydrophobic mismatch stabilizes the formation of an additional interaction. In summary, the membrane protein automata reproduce the observed membrane-dependent energetic trends, in which ΔG_{asso} decreases with increasing hydrophobic mismatch square (compare **Fig. 2h** with **Fig. 3j**), and ΔG_{diff} increases with increasing hydrophobic mismatch square (compare **Fig. 2i** and **Fig. 3k**).

The ψ_{norm} used in the automata is the average of ψ_{norm} values in all four membranes investigated. An underlying assumption in the usage of the same ψ_{norm} with different ψ/ψ_{norm} to approximate increased/decreased magnitude of deformation energy is that the relative perferences among the local-configurations hold in different cases. This is strictly not correct, given the different contributions of bending and compression to the deformation energy in the four membranes investigated as discussed in Supplementary Note 1. However, since we failed to observe significant morphological differences among the membranes, *i.e.* one local-configuration is strong favored in some membranes and strongly unfavored in the others, we think this assumption is sound at least in the our case.

References

1 Ursell, T., Huang, K. C., Peterson, E. & Phillips, R. Cooperative gating and spatial organization of membrane proteins through elastic interactions. *PLoS Comput Biol* **3**, e81, doi:10.1371/journal.pcbi.0030081 (2007).

2 Kahraman, O., Koch, P. D., Klug, W. S. & Haselwandter, C. A. Bilayer-thickness-mediated interactions between integral membrane proteins. *Phys Rev E* **93**, 042410, doi:10.1103/PhysRevE.93.042410 (2016).

3 Saad, Y. *Iterative Methods for Sparse Linear Systems*. 2nd edn, 414 (Society for Industrial and Applied Mathematics, 2003).

4 Rawicz, W., Olbrich, K. C., McIntosh, T., Needham, D. & Evans, E. Effect of chain length and unsaturation on elasticity of lipid bilayers. *Biophys J* **79**, 328-339, doi:10.1016/S0006-3495(00)76295-3 (2000).

5 Deserno, M. Fluid lipid membranes: from differential geometry to curvature stresses. *Chem Phys Lipids* **185**, 11-45, doi:10.1016/j.chemphyslip.2014.05.001 (2015).

6 Toffoli, T. & Margolus, N. *Cellular Automata Machines: A New Environment for Modeling*. (The MIT Press, 1987).
