# Supplementary material for: Membrane-mediated protein interactions drive membrane protein organization
Source: Nat Commun. 2022 Nov 30;13:7373. doi: 10.1038/s41467-022-35202-8 (PMC9712761; doi:10.1038/s41467-022-35202-8)
Supplement: Supplementary file 2 — Description of Additional Supplementary Files [file 41467_2022_35202_MOESM2_ESM.pdf]

Membrane-mediated protein interactions drive membrane protein organization

Description of Supplementary Movies

Supplementary Movie 1. High-Speed Atomic Force Microscopy movie of the experimental design to study membrane-mediated interactions (see **Fig. 1g**). Following the physisorption of the reconstituted AqpZ membranes to the mica (see **Methods**), AqpZ arrays were observed on the bare mica (~0s). The addition of lipids to the HS-AFM fluid chamber (~47s) initiated bilayer spreading and membrane fusion (~230s). During this process AqpZ molecules started to diffuse and explored the entire membrane (~290s). 100% membrane coverage was achieved after ~365s, and 100% coverage of the membrane by diffusing molecules was observed after ~415s. Imaging parameters: 1 frame/s, 1 nm/pixel.

Supplementary Movie 2. Overview High-Speed Atomic Force Microscopy movie of AqpZ diffusing in the membrane and associating/dissociating to and from arrays (see **Fig. 1i**). HS-AFM imaging reveals membrane diffusing AqpZ as transient streaks in scan lines. Imaging parameters: 1 frame/s, 1 nm/pixel.

Supplementary Movie 3. Overview High-Speed Atomic Force Microscopy movie of large AqpZ 2D-arrays, ~40 minutes after continuous bilayer formation (see **Fig. 2a**). The AqpZ 2D-arrays changed shape with local growth and contraction but without global changes in array size. Imaging parameters: 1 frame/s, 1 nm/pixel.

Supplementary Movie 4. High-Speed Atomic Force Microscopy movie of AqpZ 2D-array dynamics, ~120 minutes after continuous bilayer formation (see **Fig. 2b**). Single-molecule membrane-mediated association/dissociation dynamics to and from the AqpZ array edges were observed. Imaging parameters: 1 frame/s, 0.5 nm/pixel.

Supplementary Movie 5. High-Speed Atomic Force Microscopy movie (intermediate magnification) view of AqpZ 2D-array dynamics, ~40 minutes after continuous bilayer formation (Fig. 2c). Imaging parameters: 1 frame/s, 0.33 nm/pixel.

Supplementary Movie 6. High-Speed Atomic Force Microscopy movie (high magnification) of AqpZ 2D-array dynamics, ~15 minutes after continuous bilayer formation (see **Fig. 2d**). Imaging parameters: 1 frame/s, 0.17 nm/pixel.

Supplementary Movie 7. High-Speed Atomic Force Microscopy movie of AqpZ 2D-array dynamics at a faster imaging rate. The imaging rate was set to be faster (250ms) than the fast AqpZ dissociation time constant. Imaging parameters: 4 frame/s, 0.5 nm/pixel.

Supplementary Movie 8. Membrane protein automata (see **Extended Data Fig. 10).** Top row: simulations of AqpZ array dynamics with different hydrophobic mismatch values. Bottom row: simulations favoring micro-geometries 2-4, respectively. See **Supplementary Note 2** for a detailed description of the membrane protein automata.

Supplementary Movie 9. High-Speed Atomic Force Microscopy movie of non-tetrameric AqpZ-W14A oligomers along the 2D-array edges in C20 lipids (see **region 1, Fig. 4a**). Imaging parameters: 1 frame/s, 0.5 nm/pixel.

Supplementary Movie 10. High-Speed Atomic Force Microscopy movie of non-tetrameric AqpZ-W14A oligomers along the 2D array edges in C20 lipids (region 2, Fig. 4b). Imaging parameters: 1 frame/s, 0.5 nm/pixel.
