# Supplementary material for: Membrane-mediated protein interactions drive membrane protein organization
Source: Nat Commun. 2022 Nov 30;13:7373. doi: 10.1038/s41467-022-35202-8 (PMC9712761; doi:10.1038/s41467-022-35202-8)
Supplement: Supplementary file 13 — Reporting Summary [file 41467_2022_35202_MOESM13_ESM.pdf]

nature portfolio

Corresponding author(s): Simon SCHEURING

Last updated by author(s): Nov 17, 2022

Reporting Summary

Nature Portfolio wishes to improve the reproducibility of the work that we publish. This form provides structure for consistency and transparency in reporting. For further information on Nature Portfolio policies, see our Editorial Policies and the Editorial Policy Checklist.

Statistics

For all statistical analyses, confirm that the following items are present in the figure legend, table legend, main text, or Methods section.

n/a	Confirmed	
☐	☒	The exact sample size (n) for each experimental group/condition, given as a discrete number and unit of measurement
☐	☒	A statement on whether measurements were taken from distinct samples or whether the same sample was measured repeatedly
☒	☐	The statistical test(s) used AND whether they are one- or two-sided<br>*Only common tests should be described solely by name; describe more complex techniques in the Methods section.*
☒	☐	A description of all covariates tested
☒	☐	A description of any assumptions or corrections, such as tests of normality and adjustment for multiple comparisons
☐	☒	A full description of the statistical parameters including central tendency (e.g. means) or other basic estimates (e.g. regression coefficient) AND variation (e.g. standard deviation) or associated estimates of uncertainty (e.g. confidence intervals)
☒	☐	For null hypothesis testing, the test statistic (e.g. F, t, r) with confidence intervals, effect sizes, degrees of freedom and P value noted<br>*Give P values as exact values whenever suitable.*
☒	☐	For Bayesian analysis, information on the choice of priors and Markov chain Monte Carlo settings
☒	☐	For hierarchical and complex designs, identification of the appropriate level for tests and full reporting of outcomes
☒	☐	Estimates of effect sizes (e.g. Cohen's d, Pearson's r), indicating how they were calculated

Our web collection on statistics for biologists contains articles on many of the points above.

Software and code

Policy information about availability of computer code

Data collection	Igor Pro 7 on HS-AFM (SS-NEX, BIRM, Tsukuba, Japan)
Data analysis	MATLAB R2021b, ImageJ 1.53h, customized codes (available from the corresponding author upon reasonable request)

For manuscripts utilizing custom algorithms or software that are central to the research but not yet described in published literature, software must be made available to editors and reviewers. We strongly encourage code deposition in a community repository (e.g. GitHub). See the Nature Portfolio guidelines for submitting code & software for further information.

Data

Policy information about availability of data

All manuscripts must include a data availability statement. This statement should provide the following information, where applicable:

- Accession codes, unique identifiers, or web links for publicly available datasets
- A description of any restrictions on data availability
- For clinical datasets or third party data, please ensure that the statement adheres to our policy

All data supporting the findings of this study are available within the article and its supplementary information files. Additional information, relevant raw data, and customized codes on data analysis and simulation are available from the corresponding author upon reasonable request and at the earliest convenience.

Human research participants

Policy information about studies involving human research participants and Sex and Gender in Research.

Reporting on sex and gender	N/A
Population characteristics	N/A
Recruitment	N/A
Ethics oversight	N/A

Note that full information on the approval of the study protocol must also be provided in the manuscript.

Field-specific reporting

Please select the one below that is the best fit for your research. If you are not sure, read the appropriate sections before making your selection.

☒ Life sciences ☐ Behavioural & social sciences ☐ Ecological, evolutionary & environmental sciences

For a reference copy of the document with all sections, see nature.com/documents/nr-reporting-summary-flat.pdf

Life sciences study design

All studies must disclose on these points even when the disclosure is negative.

Sample size	No statistical methods were used to determine sample size. For AqpZ array analysis, at least 80 events (in most cases several hundred events) were used for fitting for each array. This number is sufficient for the exponential fittings applied. The event number (sample size) generally depends on the imaging conditions, e.g. movie length, imaging and tip quality etc.. Only movies of high quality, similar to the examples given in the main text, and of significant lengths, >100 frames (1 frame/s), were analyzed. This criteria ensures that sufficient amount of events were analyze. For HS-AFM height spectroscopy, one second of data (height-time trace) usually provides hundreds of molecular diffusing events, given the high sampling rate ~600 kHz. In this study, all the height-time traces were > 5 seconds. Thus, several hundreds of events were acquired for diffusion events analysis, sufficient for the exponential fitting applied. For HS-AFM imaging of non-tetramer WT AqpZ, since these species were rare, we did not report any statistics.
Data exclusions	For HS-AFM imaging and AqpZ array analysis, all the arrays analyzed were of similar quality as the data shown in figure 2b and supplementary movie 4, where single molecules were clearly resolved. In occasional case, dissociation events were omitted due to a single poor quality frame, where the molecular environment was hard to be determined. In addition, extremely long events (<1%) were omitted from event dwell time fittings. For HS-AFM height spectroscopy (HS-AFM-HS), the height-traces with manageable background noise were processed and analyzed. The standards for HS-AFM-HS data processing and analysis were well established in previous studies (see Methods).
Replication	Comparable data, including both movies (HS-AFM imaging) and height-time traces (HS-AFM height spectroscopy), in all conditions was recorded with the same reconstitution sample and at different imaging areas and different days. Data was taken with different tips but all analyzed data was of high quality, similar to the examples shown in the main figures. Three replicas in each condition were performed for error estimates.
Randomization	Randomization is not applicable to our biophysical experiments. This study did not allocate experimental/control groups.
Blinding	Blinding is not applicable. This is an experimental biophysics study, combined with quantitative analysis. For the data analysis, we developed an objective analysis workflow, as described in the supplementary information, and implemented this workflow using customized codes. All data were analyzed using the same computational methods.

Reporting for specific materials, systems and methods

We require information from authors about some types of materials, experimental systems and methods used in many studies. Here, indicate whether each material, system or method listed is relevant to your study. If you are not sure if a list item applies to your research, read the appropriate section before selecting a response.

Materials & experimental systems

n/a	Involved in the study
☒	☐ Antibodies
☒	☐ Eukaryotic cell lines
☒	☐ Palaeontology and archaeology
☒	☐ Animals and other organisms
☒	☐ Clinical data
☒	☐ Dual use research of concern

Methods

n/a	Involved in the study
☒	☐ ChIP-seq
☒	☐ Flow cytometry
☒	☐ MRI-based neuroimaging
